# Quality of reporting of drug exposure in pharmacoepidemiological studies

**DOI:** 10.1002/pds.5020

**Published:** 2020-05-11

**Authors:** Mirjam Hempenius, Kim Luijken, Anthonius de Boer, Olaf Klungel, Rolf Groenwold, Helga Gardarsdottir

**Affiliations:** ^1^ Division of Pharmacoepidemiology and Clinical Pharmacology, Utrecht Institute for Pharmaceutical Sciences Utrecht University Utrecht the Netherlands; ^2^ Department of Clinical Epidemiology Leiden University Medical Centre Leiden the Netherlands; ^3^ Julius Center for Health Sciences and Primary Care University Medical Center Utrecht Utrecht the Netherlands; ^4^ Department of Biomedical Data Sciences Leiden University Medical Center Leiden the Netherlands; ^5^ Department of Clinical Pharmacy, Division Laboratories, Pharmacy and Biomedical Genetics University Medical Center Utrecht Utrecht the Netherlands; ^6^ Faculty of Pharmaceutical Sciences University of Iceland Reykjavik Iceland

**Keywords:** drug exposure, pharmacoepidemiology, reporting, systematic review

## Abstract

**Purpose:**

Exposure definitions vary across pharmacoepidemiological studies. Therefore, transparent reporting of exposure definitions is important for interpretation of published study results. We aimed to assess the quality of reporting of exposure to identify where improvement may be needed.

**Method:**

We systematically reviewed observational pharmacoepidemiological studies that used routinely collected health data, published in 2017 in six pharmacoepidemiological journals. Reporting of exposure was scored using 11 items of the ISPE‐ISPOR guideline on reporting of pharmacoepidemiological studies.

**Results:**

Of the 91 studies included, all studies reported the type of exposure (100%), while most reported the exposure risk window (85%) and the exposure assessment window (98%). Operationalization of the exposure window was described infrequently: 16% (14/90) of the studies explicitly reported the presence or absence of an induction period if applicable, 11% (5/47), and 35% (17/49) reported how stockpiling and gaps between exposure episodes were handled, respectively, and 35% (17/49) explicitly mentioned the exposure extension. Switching/add‐on was reported in 62% (50/81). How switching between drugs was dealt with and specific drug codes were reported in 52 (57%) and 24 (26%) studies, respectively.

**Conclusion:**

Publications of pharmacoepidemiological studies frequently reported the type of exposure, the exposure risk window, and the exposure assessment window. However, more details on exposure assessment are needed, especially when it concerns the operationalization of the exposure risk window (eg, the presence or absence of an induction period or exposure extension, handling of stockpiling and gaps, and specific codes), to allow for correct interpretation, reproducibility, and assessment of validity.

**KEY POINTS**

- Transparent reporting of exposure definitions is important for interpretation and reproducibility of published study results.
- Publication guidelines, like RECORD‐PE and the joint ISPE‐ISPOR guideline, provide guidance to researchers to present their research in a transparent and complete manner.
- This systematic review showed that publications of pharmacoepidemiological studies frequently reported the type of exposure, the exposure risk window, and the exposure assessment window. However, more details on exposure assessment are needed, especially when it concerns the operationalization of the exposure risk window, to allow for correct interpretation, reproducibility, and assessment of validity.

## 1 INTRODUCTION

Transparent reporting is important for interpretation of published study results, but also for reproducibility and validity assessment. Reporting guidelines support researchers to describe their research in a transparent and complete manner, like CONSORT,^1^ for reporting on clinical trials, STROBE,^2^ for reporting on observational studies, and STROBE‐RECORD,^3^ for reporting on observational studies using routinely collected health data. These guidelines however do not capture the complex operational details required for the conduct of pharmacoepidemiological research, including complex exposure ascertainment algorithms.^4^ Recently, two guidelines were published that focus on these complex operational details. The first is a guideline of a joint ISPE‐ISPOR Task Force,^5^ published in 2017 and the second an extension of the RECORD statement, the RECORD‐PE,^4^ published in 2018.

Both RECORD‐PE and the joint ISPE‐ISPOR Task‐Force guidelines include a separate section on the reporting of the drug exposure definition, with the latter most specific on operational details of exposure ascertainment. Reporting details about the drug exposure definition is important, since drug exposure can be defined in various ways in observational research, including time‐fixed, time‐varying, and cumulative drug exposure definitions. In particular, regarding time‐varying definitions, researchers must make choices how the drug exposure risk window is defined and how gaps or overlapping periods between drug prescriptions or dispensings are being addressed when constructing drug use episodes. As different choices may lead to different effects being estimated,^6^, ^7^, ^8^, ^9^ it is important that researchers report transparently how exposure was defined to aid correct interpretation of results.

It takes a substantial amount of time to see the effects of published guidelines on transparent reporting in practice. In the case of CONSORT, reporting has improved in the 20 years after the first version was published, but remains suboptimal, with on average 18 of 37 items being reported over the period of 2010‐2014.^10^, ^11^ Also in case of STROBE, reporting has improved after publishing of the guideline, but there is still room for improvement as the median compliance with the 22 items is 77% in 2016, 9 years after STROBE was published.^12^ We therefore assessed the quality of exposure assessment reporting according to ISPE‐ISPOR Task Force Guidelines, in studies published around the time frame where these guideline were published, to provide a baseline exposure assessment and to determine where improvement may be needed.

## 2 METHODS

To assess the quality of reporting of pharmacoepidemiological research, we systematically reviewed observational pharmacoepidemiological studies that used routinely collected health data. We used the guideline by the ISPE‐ISPOR Task Force to evaluate quality of reporting of exposure, because this guideline aims to facilitate not only validity assessment but also (direct) reproducibility, and therefore is most specific about operationalization of the exposure risk window.

### 2.1 Journal selection and eligibility of studies

We selected six pharmacoepidemiological journals: *Annals of Pharmacotherapy, British Journal of Clinical Pharmacology, Drug Safety, European Journal of Clinical Pharmacology, Pharmacoepidemiology and Drug Safety, and Pharmacotherapy*. This selection of journals was based on predefined criteria: at least 20 hits in 2017 that met the search criterion in search of routinely collected health data. These 20 hits had to cover at least 5% of the total publications of that specific journal and the journal had to be classified in the category “Pharmacology and pharmacy” at InCites Journal Citation Reports,^13^ with an impact factor of at least 2^13^ (details in file S1 in Data S1).

We included all of the studies published in 2017 in these six journals that used routinely collected health data for exposure assessment, such as prescription data, dispensing data or claims data. All studies needed to include at least 250 subjects, to ensure that the exposure assessment was not performed manually. Studies that used questionnaires for exposure assessment were excluded. Studies assessing vaccines were also excluded, as our interest was in reporting of exposure that is used over a certain period of time, whereas vaccines are administered as single administrations.

### 2.2 Extraction of study characteristics

The following general items were extracted: journal name, word count limit of the article (≤1500 [short report], 1500‐3000, 3000‐4000 or ≥4000 words), study design (cohort, case‐control, case‐crossover or other study design), the route of administration of the drug (oral/inhaled or intravenous/subcutaneous), the type of outcome (beneficial or adverse effect), the number of included subjects (categorized as 250‐1000, 1001‐10 000, 10 001‐100 000 and >100 000 subjects), the type of database used for assessment of exposure (claims, General Practitioner [GP], pharmacy or hospital database), and the geographical area where the study was conducted, defined as continents.

**FIGURE 1:**
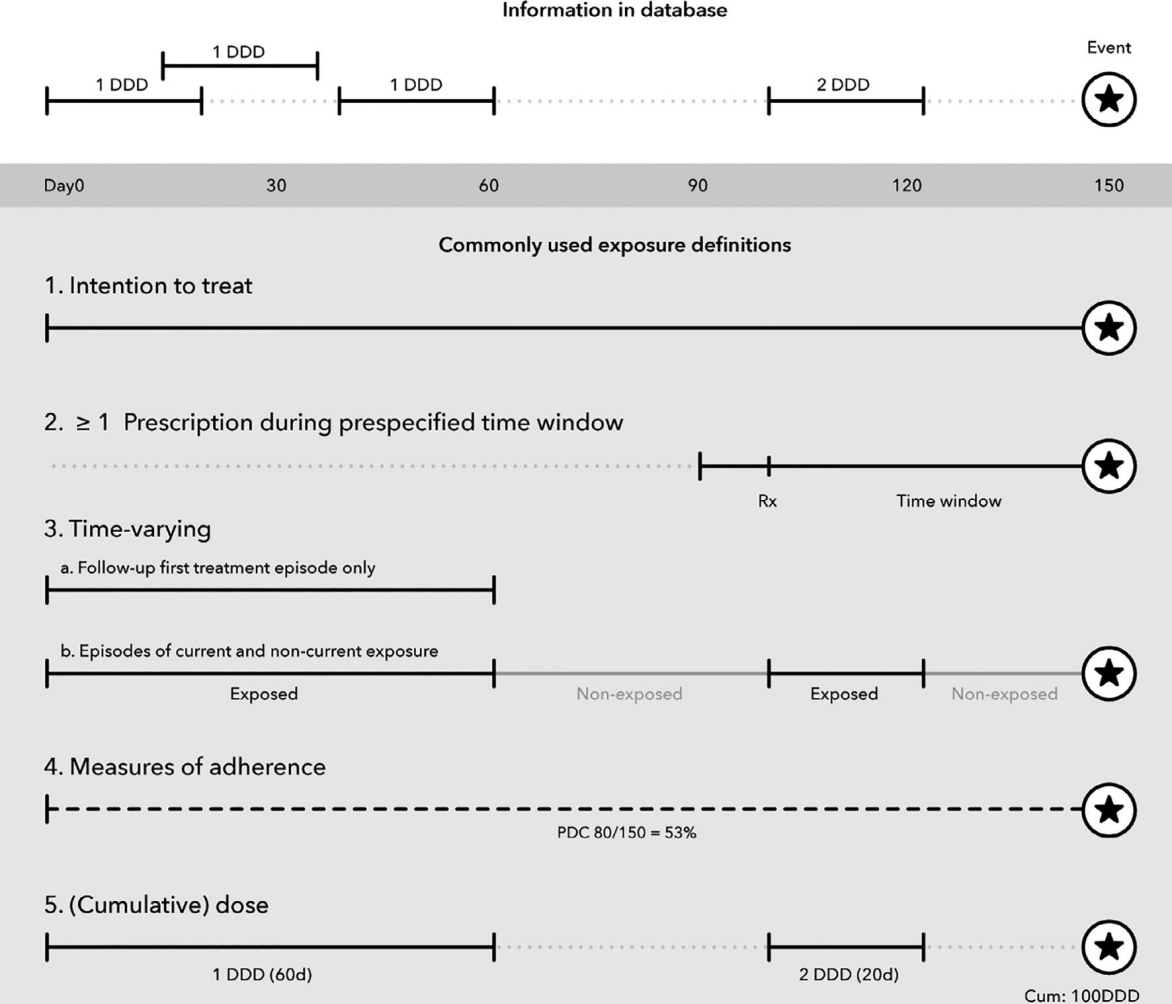
Categorization of commonly used exposure definitions in pharmacoepidemiological studies. Different types of exposure definition are applied in pharmacoepidemiological research. We divided these in five categories for further analysis: 1. intention to treat: exposure at baseline is included as a time‐fixed variable in the model; 2. the presence of ≥1 prescriptions during a certain time period, for example during pregnancy or during the last 12 months prior to the event; 3. time‐varying: episodes of (non)exposure are constructed based on duration of each prescription; 4. measures of adherence: for example, level of exposure is measured as proportion of days covered and 5. dose and cumulative dose: exposure is modeled as a continuous or ordinal variable and the effects of different dosages are compared (time‐fixed or time‐varying). DDD, daily defined dose; PDC, percentage of days covered; Rx, prescription

Because the ISPE‐ISPOR guideline focuses on time‐varying exposure, we categorized the studies according to exposure definition in five predefined categories (illustrated in Figure 1):

1. Intention to treat: drug exposure at baseline was included as a time‐fixed variable in the model.
2. The presence of ≥1 prescription during a certain period, for example during pregnancy or during the last 12 months prior to the outcome event of interest.
3. Time‐varying: episodes of (non)exposure were constructed based on duration of each prescription, without distinguishing between different dosages.
4. Measures of adherence: for example, level of drug exposure was measured as proportion of days a subject has drug in possession divided by the total number of days of follow‐up.
5. Dose and cumulative dose: drug exposure was modeled as a continuous or ordinal variable and the effects of different dosages at index date were compared.

This categorization was carried out to notice any difference in reporting between studies with different types of exposure definitions. Characteristics of each included study were extracted independently by two reviewers (M.H. and K.L.).

### 2.3 Evaluation of reporting quality

Quality of reporting of exposure was assessed according to the ISPE‐ISPOR guideline.^5^ All items listed under “Section D – Reporting on exposure definition” were assessed. Item D4 of the guideline contains four elements (“Codes, frequency and temporality of codes, diagnosis position and care setting”) and is linked with guideline section C (“Inclusion and exclusion criteria”) for further clarification. In this section these items are included as separate items, so we decided to split D4 into four separate items as well. The item “diagnosis position (D4)” was excluded from the final list of items as we considered this item not to be relevant for drug exposures. The resulting 11 items are listed in Table 1.

**TABLE 1:** Items pertaining to the quality of reporting of exposure definition in pharmacoepidemiological research. These items are selected from the ISPE‐ISPOR Joint Task Force guideline^5^

Item	Explanation	ISPE‐ISPOR item
1. Type of exposure	The type of exposure that is captured or measured, for example, drug vs procedure, new use, incident, prevalent, cumulative, time‐varying.	D1
2. Exposure risk window (ERW)	The ERW is specific to an exposure and the outcome under investigation. For drug exposures, it is equivalent to the time between the minimum and maximum hypothesized induction time following ingestion of the molecule.	D2
3. Induction period	Days on or following study entry date during which an outcome would not be counted as “exposed time” or “comparator time.”	D2a
4. Stockpiling	The algorithm applied to handle leftover days' supply if there are early refills.	D2b
5. Bridging exposure episodes	The algorithm applied to handle gaps that are longer than expected if there was perfect adherence (eg, non‐overlapping dispensation + day's supply).	D2c
6. Exposure extension	The algorithm applied to extend exposure past the days' supply for the last observed dispensation in a treatment episode.	D2d
7. Switching/add on	The algorithm applied to determine whether exposure should continue if another exposure begins.	D3
8. Codes	The exact drug, diagnosis, procedure, lab or other codes used to define inclusion/ exclusion criteria.	D4
9. Frequency and temporality of codes	The temporal relation of codes in relation to each other as well as the study entry date (SED). When defining temporality, be clear whether or not the SED is included in assessment windows (eg, occurred on the same day, 2 codes for A occurred within 7 d of each other during the 30 d prior to and including the SED).	D4
10. Care setting	The restrictions on codes to those identified from certain settings, for example, inpatient, emergency department, nursing home.	D4
11. Exposure assessment window (EAW)	A time window during which the exposure status is assessed. Exposure is defined at the end of the period. If the occurrence of exposure defines cohort entry, for example, new initiator, then the EAW may be a point in time rather than a period. If EAW is after cohort entry, follow‐up window must begin after EAW.	D5

To ensure uniform interpretation of the listed items when assessing the articles, eight randomly chosen articles were reviewed independently by two reviewers (M.H. and K.L.) and discrepancies of the scores were discussed. This resulted in a formalized data extraction form that was used for the remaining articles.

Of each reviewed article, we scrutinized the methods sections and, if referenced to, the supplementary materials for the data extraction. Each of the 11 items was scored as “not reported,” “reported,” or “not applicable.” An item could be scored as “not applicable” if this item was not relevant for that specific study. For example, if exposure was defined as receiving 1 or more drug prescriptions, it was not relevant to report how stockpiling and handling gaps were dealt with. The items 3 (induction period, D2a) and 6 (exposure extension, D2d) (see Table 1) could also be mentioned implicitly. For example, if an author stated that “the follow‐up started on the day of the first prescription and ended after the duration of the last prescription,” it is implicit that there was no induction period and no extension of the exposure risk window. For items 3 and 6 we therefore also scored whether reporting was “explicit” or “implicit.”

All articles were reviewed independently by two reviewers (M.H. and K.L.) and discrepancies were discussed until consensus was reached. The interobserver agreement (kappa) was 0.53. The eight studies that were used to refine the assessment tool were excluded from the calculation of the kappa.

In addition to the 11 ISPE‐ISPOR items, we assessed whether the exposure definition was accompanied by a figure for graphical representation, since this is recommended in the ISPE‐ISPOR guideline for study design in general (item B1).

### 2.4 Data analysis

For each of the ISPE‐ISPOR reporting items, the primary outcome was the percentage of articles that reports this item, where applicable. The results were stratified by study design, the number of patients included, the type of outcome, the route of administration, the exposure definition, the type of database used, and word limit of the article. The percentage of studies that included a graphical presentation of the exposure definition was considered as a secondary outcome. In the case of multiple types of exposure, designs or types of databases within one publication, we analyzed them as one unit within the main analysis, including all information that was mentioned in the publication. For stratification purposes, we only used the information provided for that specific design, exposure type or database.

## 3 RESULTS

### 3.1 Selection and characteristics of studies

**FIGURE 2:**
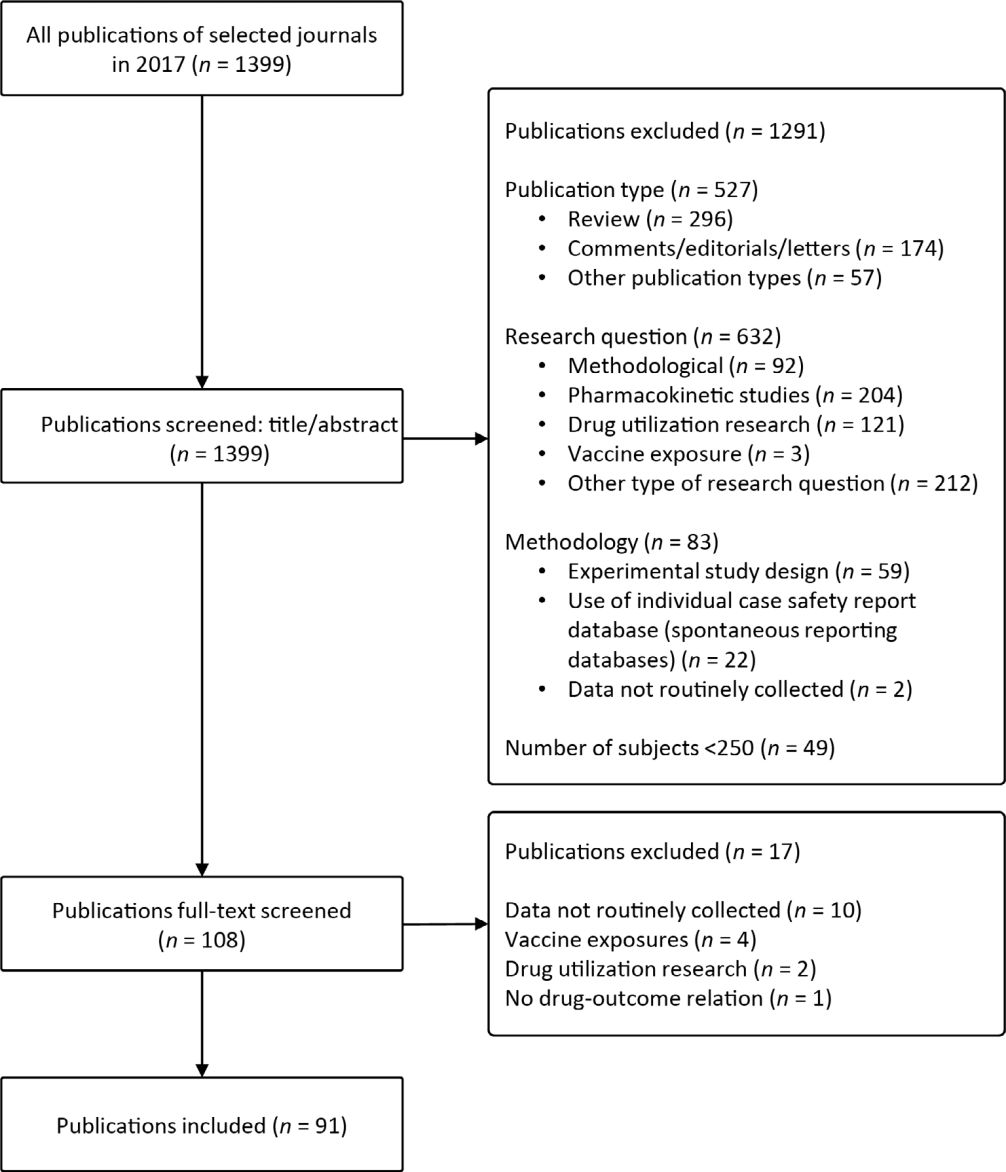
Flow chart of the search and screening process to select pharmacoepidemiological studies using routinely collected data. All articles published in 2017 in the following six journals were included in the first step: Annals of Pharmacotherapy, British Journal of Clinical Pharmacology, Drug Safety, European Journal of Clinical Pharmacology, Pharmacoepidemiology and Drug Safety, and Pharmacotherapy

A total of 91 articles were included (Figure 2, see file S2 for all references in Data S2). The characteristics of all 91 articles are summarized in Table 2. Different types of exposure were applied; 24 (26%) studies performed an intention to treat analysis, 43 (47%) studies applied a time‐varying exposure assessment; 19 (21%) studies assessed the occurrence of one or more prescriptions during a certain period, 4 (4%) used measures of adherence as exposure, and 3 (3%) investigated the effect of (cumulative) dose. Two studies applied multiple definitions in their study.

**TABLE 2:** Characteristics of the studies included for evaluation of quality of reporting of pharmacoepidemiological studies (n = 91)

	n (%)
**Journal**	
Annals of Pharmacotherapy	7 (8)
British Journal of Clinical Pharmacology	16 (18)
Drug Safety	8 (9)
European Journal of Clinical Pharmacology	17 (19)
Pharmacoepidemiology and Drug Safety	27 (30)
Pharmacotherapy	16 (18)
**Design** ^a^	
Cohort	64 (70)
Case‐control	25 (28)
Case‐crossover	4 (4)
**Type of outcome**	
Beneficial effects	18 (20)
Adverse effects	67 (74)
Beneficial and adverse effects	6 (7)
**Number of subjects included**	
250‐1000	13 (14)
1001‐10 000	30 (33)
10 001‐100 000	24 (26)
>100 000	24 (26)
**Type of database** ^a^	
Claims database	41 (44)
GP database	17 (19)
Hospital database	18 (20)
Pharmacy database	16 (18)
Unclear	2 (2)
**Geographical area** ^a^	
Europe	39 (43)
Asia	15 (17)
North America	36 (40)
Australia	2 (2)
**Route of administration**	
Oral and inhaled	80 (88)
Intravenous and subcutaneous	11 (12)
**Exposure definition** ^a^	
Intention to treat	24 (26)
≥1 prescription/dispense during a certain period	19 (21)
Time‐varying	43 (47)
Measures of adherence	4 (4)
(Cumulative) dose at index date	3 (3)

a Sum of n may exceed 91.

### 3.2 Reporting quality

An average of 6.6 (SD 1.8) items were reported out of the 11 items pertaining to quality of reporting of exposure definition. The median number of items reported was 7, ranging between 2 and 10 per study. The reporting of each item is presented in Table 3. Most studies reported the type of exposure (eg, current use, cumulative dose) (n = 91, 100%), the exposure risk window in general terms (n = 77, 85%), and the exposure assessment window (n = 89, 98%). The operationalization of the exposure window was infrequently described: of 90 studies that should report on an induction period, 14 (16%) studies explicitly reported the presence or absence of an induction period, and another 67 (74%) reported this implicitly. Among the 49 studies where exposure extension was possible, 17 (35%) studies reported explicitly how long the exposure was extended and 10 (20%) studies mentioned this implicitly. Stockpiling and bridging of exposure episodes was reported in 5 of 47 (11%) and 18 of 44 (41%) studies. How switching between drugs or add‐on was dealt with was reported in 50 of 81 (62%) studies where this item was applicable. Specific drug codes and care setting were reported in 24 of 91 (26%) and 67 of 91 (74%) studies. Temporality of codes was reported in 77 of 91 (85%) studies.

**TABLE 3:** Quality of Reporting of exposure for the included studies. For each specific item, the number of studies reporting that item is shown. (n = 91**)**

Item	Studies, n^b^	Reported, n (%)
1. Type of exposure	91	91 (100)
2. Exposure risk window (ERW)	91	77 (85)
3. Induction period^a^	90	81 (90)
Explicit		14 (16)
Implicit		67 (74)
4. Stockpiling	47	5 (11)
5. Bridging exposure episodes	44	18 (41)
6. Exposure extension^a^	49	27 (55)
Explicit		17 (35)
Implicit		10 (20)
7. Switching/ add on	81	50 (62)
8. Codes	91	24 (26)
9. Frequency and temporality of codes	91	77 (85)
10. Care setting	91	67 (74)
11. Exposure Assessment Window (EAW)	91	89 (98)

a When explicitly mentioning an induction period, a period after the index date is clearly excluded in the exposure risk window. Stating that follow‐up started on the day of the first prescription implies implicitly that there was no induction period. The same reasoning applies to the extension period.

b Total number of studies to which this item was applicable.

Eleven studies (12%) supported the reporting of their exposure definition with a graphical representation, nine of them in the article itself and two in the supplementary materials.

### 3.3 Stratification by study characteristics

The exposure definition determined which details needed to be reported regarding the exposure assessment. Stratification by exposure definition showed that studies using time‐varying definitions report on average more items compared with all other definitions (7.4 (SD 1.7) vs 6.0 (SD 1.7), Table S1 in Data S3). The items stockpiling (item 4) and handling gaps (item 5) were considered to be relevant only for the time‐varying definitions, where they were reported in 7% and 42% of the studies respectively. Exposure extension (item 6) was also reported more often in the studies with a time‐varying exposure assessment (42%) vs studies with another exposure definition (6%).

Stratification by route of administration also showed differences, studies on intravenous or subcutaneous administered drugs reported less frequently on nearly all items than studies on oral or inhaled drugs.

Stratification by study design, number of subjects included in the study, type of outcome, type of database used, and word limit of the article, did not reveal major differences. The results of the stratified analyses are available in the file S3, Tables S1‐S7 in Data S3.

## 4 DISCUSSION

This systematic review of quality of reporting of drug exposure in pharmacoepidemiological studies showed that none of the studies assessed met all requirements of reporting of drug exposure as defined by the ISPE‐ISPOR guideline. The number of reported items varied widely between studies, ranging from 2 to 10. In general, the conceptual details about the exposure risk window and the exposure assessment window were reported relatively often (85% and 98%, respectively). However, the operational details concerning the construction of the exposure risk window were reported less often. For example, handling gaps and overlapping episodes were reported in only 11% and 41% of studies, where this type of reporting was applicable, thereby impeding reproducibility.

Our findings on the substandard quality of reporting of pharmacoepidemiological database studies are in line with the results of a study by Wang et al.^14^ In their attempt to reproduce 31 pharmacoepidemiological database studies, they noted that code lists for outcomes, covariates and inclusion/exclusion criteria were reported in only 11 of 31 studies (35%). Likewise, we found that code lists for drug exposure were only reported in 24 of 91 studies (26%). Although not all details were reported, Wang et al were able to reproduce several database studies with high accuracy, but mention that this was partly due to “*the efforts of the reproduction team, a group of pharmacoepidemiologists with decades of experience, to make informed guesses regarding variable definitions or other key decisions when these were not clearly specified in the original articles*.”^14^ It is debatable whether this level of expertise could be expected from the general reader of pharmacoepidemiological studies. Therefore, it is important that details are reported clearly for correct interpretation and reproducibility of study results.

Besides reporting operational details, it is important to clearly describe the choices regarding the exposure definition, such as the exposure risk window and what type of exposure is examined. Currently there are a number of guidelines to support these methodological choices, like the ISPE Guidelines for good pharmacoepidemiology practices,^15^ the ENCePP Guide on Methodological Standards in Pharmacoepidemiology,^16^ the FDA guidance for Conducting and Reporting Pharmacoepidemiologic Safety Studies Using Electronic Healthcare Data,^17^ the User's Guide for Developing a Protocol for Observational Comparative Effectiveness Research by the Agency for Healthcare Research and Quality,^18^ and the EU Good Pharmacovigilance Practice (Module VIII).^19^ Without clear reporting of all key decisions, assessment of the validity of the results will be difficult.

A strength of this study was the independent assessment of all studies by two researchers. This also revealed one of the limitations of this study: substantial interpretation was needed by the researchers to score all studies, which is also reflected in a moderate kappa of 0.53. This could partially be explained by the fact that not all questions of the checklist applied to each study included in our study. The listed guideline items could be scored most easily for a study design with time‐varying treatment episodes. The items 4 (stockpiling, D2b), 5 (handling gaps, D2c), 6 (exposure extension, D2d) and 7 (switching, D3) were for other types of exposure, (eg, intention to treat) not relevant and thus scored NA. This is also reflected in the kappa and the percentage agreement of these items (file S4 in Data S4). When we recalculated kappa, with only a contrast between reporting something (Yes) or not (No or NA), this resulted in a kappa of 0.64.

There was also a difference between the reporting of exposure to drugs that were oral or inhaled administered, compared with the reporting of exposure drugs that were intravenous or subcutaneous administered. This might be explained by the fact that intravenous or subcutaneous drugs are commonly identified by procedural codes instead of drug dispensing information. These data contain other information about the drug exposure, resulting in also another way of reporting of the drug exposure assessment, which might not be captured in the guideline used for this review.

Another possible limitation concerns the inclusion of only publications in six pharmacoepidemiological journals. The results may thus not be generalizable to the quality of reporting of drug exposure in general. Furthermore, we only searched the methods and supplementary materials (if referenced) for exposure assessment information, possibly missing out on information described in other sections of the publication. For transparency reasons, it is however still recommended to describe all methodological choices in the methods section. In addition, it might also be possible that these details are described in other study reports, such as reports provided to the regulator, but are left out of the publication, due to word count limitations. We did, however, not see differences in results between publications in journals with a strict word limit (≤3000 words) compared to publications in journals with less stringent word limits.

Suboptimal reporting is not unique to pharmacoepidemiological research and the effort for more transparent reporting has facilitated the development of various reporting guidelines, such as CONSORT and STROBE. To further stimulate use of these, endorsement by many journals has resulted in improved reporting, but after two decades, adherence to CONSORT is still suboptimal.^20^, ^21^, ^22^ In order to accelerate adherence to RECORD‐PE and the ISPE‐ISPOR guideline, it might be considered to oblige authors to use one of these two guidelines. Four of the six included journals (*Annals of Pharmacotherapy, British Journal of Clinical Pharmacology, Drug Safety, Pharmacoepidemiology and Drug Safety*) currently recommend authors to adhere to the guidelines available through the EQUATOR network, including the RECORD‐PE guideline. One journal (*European Journal of Clinical Pharmacology*) advises to adhere to CONSORT for observational research and one journal (*Pharmacotherapy*) does not recommend a specific reporting guideline. In addition, current good practices can be used as examples. We summarized some good practices of clear reporting of exposure assessment in Textbox 1, which can be helpful for future studies. We also noticed that giving arguments for specific choices was helpful for the interpretation of the conceptual choices, as was the inclusion of a graphical representation for the interpretation of the operational choices.

**Table:** 

TEXTBOX 1 Examples of good practices for each of the items in the ISPE‐ISPOR checklist cited from included articles	
1. Type of exposure	“Those who filled a prescription for an antidepressant during this period <1 January 2007 and 31 December 2013> with no such fills during the preceding year were considered treatment initiators.”^23^
	“In the first model, the mutually exclusive binary indicators of use for each NSAID were (a) current use on the index date, (b) recent use 1 to 30 days ago, (c) past use 31 to 180 days ago, or (d) no use in the last 180 days before the index date.”^24^
2. Exposure risk window (ERW)	“For each patient, we defined a period of continuous drug use beginning with the first prescription after their 66th birthday and ending with death, discontinuation of treatment, the end of the study period (31 March 2014), or 90 days of follow‐up, whichever occurred first. <…> We based our selection of a 90‐day observation window on existing literature describing heart failure and edema within a few months of pregabalin therapy.”^25^
	“The primary outcome was hospitalization for COPD or pneumonia within 30 days after the index date <…>”^26^
3. Induction period	“<…> the effect of insulin on chronic complications may take some time, so we conducted a lag‐time analysis, whereby patients with chronic complication events that occurred 3 years after the initiation of insulin were excluded.”^27^
	“Outcomes were collected starting 30 days following the index date to ensure that events occurring during the baseline period were not mistakenly captured as study period events.”^28^
4. Stockpiling	“For overlapping prescriptions, the individual was assumed to have completed the former one before starting the second.”^29^
	“To account for gaps and overlaps in redemptions due to incomplete adherence or lost prescriptions, we presumed that health‐insured persons have drug stocks lasting up to 15 days due to incomplete compliance (‘15‐ day rule’), added apparent overlaps up to a maximum overlap duration corresponding to 25% of the quantity of the last overlapping prescription, and applied common recommendations to fill apparent gaps between prescriptions using prospective filling.”^30^
5. Bridging exposure episodes	“Discontinuation of use <was> defined as a 60‐day gap between the end of one COC prescription and the next COC prescription”^31^
6. Exposure extension	“Observation was extended by half the days supplied from the final prescription to capture outcomes that may have prompted cessation of therapy”^25^
7. Switching/ add on	“If a patient switched from warfarin to rivaroxaban or vice versa during the study period, that was considered discontinuation of the index drug, and they were censored at that time.”^32^
	“To assess whether associations varied with different antidepressants, we categorized antidepressants into 3 types (SSRI monotherapy, non‐SSRI monotherapy, or both SSRI and non‐SSRI antidepressants).”^33^
8. Codes	“Antihypertensive drugs studied were: angiotensin‐converting enzyme (ACE) inhibitors: ATC code C09A and C09B, angiotensin receptors blockers (ARBs): ATC code C09C and C09D, calcium channel blockers (CCBs): ATC code C08, β‐blockers: ATC code C07, diuretics: ATC code C03 (thiazide or thiazide‐like diuretics, loop diuretics and potassium‐sparing diuretics) and miscellaneous antihypertensive agents: ATC code C02.”^34^
	“We selected all patients who had ever received Fz/Cz <…> according to the Anatomical Therapeutic Chemical (ATC) codes N07CA03 (for Fz) and N07CA02 (for Cz) <…>.”^35^
9. Frequency and temporality of codes	“All patients included in the Cohort were followed from the 90th day after the incident ACS occurrence (index date) until the incidence of a major adverse cardiac event (MACE), death, date removed from the database or 31 December 2013, whichever came first.”^36^
10. Care setting	“Because of the high patient pharmacy loyalty in the Netherlands, the prescription records for each patient in the database are virtually complete, except for over‐the‐counter (OTC) drugs and drugs dispensed during hospitalization.”^37^
11. Exposure Assessment Window (EAW)	“As diagnosis and treatment start may be registered in different days <…>, we allowed a time interval of ±3 months from diagnosis date and start of treatment.”^38^
	“We conducted a <study of residents> prescribed digoxin at any time between 1 January 1994 and 31 December 2012, the last date for which complete data were available.”^39^

To conclude, we recommend that publications of pharmacoepidemiological studies should include more details on exposure ascertainment, especially about the operationalization of the exposure risk window (eg, the presence or absence of an induction period or exposure extension, handling of stockpiling and gaps, and specific codes), to allow correct interpretation of the results and to enable reproducibility, and validity assessment. Authors, reviewers, and editors are encouraged to pay more attention to adhere to relevant reporting guidelines such as the ISPE‐ISPOR and RECORD‐PE guidelines.

## ETHICS STATEMENT

The authors state that no ethical approval was needed.

## CONFLICT OF INTEREST

No authors report any conflict of interest.

## Supporting information


**Data S1.** Supporting Information.

**Data S2.** Supporting Information.

**Data S3.** Supporting Information.

**Data S4.** Supporting Information.
