# Supplementary material for: Quality of reporting of drug exposure in pharmacoepidemiological studies
Source: Pharmacoepidemiol Drug Saf. 2020 May 11;29(9):1141–50. doi: 10.1002/pds.5020 (PMC7539966; doi:10.1002/pds.5020)
Supplement: Supplementary file 1 — Data S1. Supporting Information. [file PDS-29-1141-s001.docx]

# Supplementary materials – selection of journals (S1)

Selection based on the following characteristics:

1. At least 20 hits obtained within 2017 with the next search strategy: *(pharmacoepidemiology OR pharmacy claims OR pharmacy data OR dispensing data OR computerized data OR computerized database OR administrative claims OR prescription claims OR prescription database OR prescription data OR health database OR health care database OR health care claims OR insurance plan OR Medicaid OR managed care Organization OR Veterans Affairs ) AND (effectiveness OR efficacy OR side-effects OR safety OR unintended effects) AND English [language]*

(based on Andrade, Kahler, Frech, & Chan, 2006)

1. Ratio of the number of publications found with this strategy and the total number of publications > 0.05 *(Note: some journals had> 20 publications with this search strategy, but compared to the total number of publications, this was only a small part. That is why we only searched for journals with a "significant" (> 5%) share of relevant hits)*
2. Categorized in journal category Pharmacology and pharmacy at InCites Journal Citation Reports.
3. Impact Factor >2 (Journal Citation Reports)

This results in the following set of journals: Annals of Pharmacotherapy, British Journal of Clinical Pharmacology, Drug Safety, European Journal of Clinical Pharmacology, Pharmacoepidemiology and Drug Safety, and Pharmacotherapy (see Figure S1) In 2017, this resulted in 1399 articles.

**Figure S1. Flow chart of the search and screening process to select journals**

8577 hits in 2357 unique journals

65 journals with ≥20 publications found with search strategy

34 journals with ≥5% relevant publications of total publications

10 journals categorized as ‘Pharmacology and pharmacy’

6 journals with impact factor ≥2:

- Annals of Pharmacotherapy
- British Journal of Clinical Pharmacology
- Drug Safety
- European Journal of Clinical Pharmacology
- Pharmacoepidemiology and Drug Safety
- Pharmacotherapy

2357 unique journals

2290 journals with <20 publications found with search strategy

31 journals with <5% relevant publications of total publications

24 journals not categorized as ‘Pharmacology and pharmacy’

4 journals with impact factor <2
