# Supplementary material for: Quality of reporting of drug exposure in pharmacoepidemiological studies
Source: Pharmacoepidemiol Drug Saf. 2020 May 11;29(9):1141–50. doi: 10.1002/pds.5020 (PMC7539966; doi:10.1002/pds.5020)
Supplement: Supplementary file 2 — Data S2. Supporting Information. [file PDS-29-1141-s002.docx]

# Supplementary materials – list of all included studies (S2)

1. Admassie E, Chalmers L, Bereznicki LR. Bleeding-related admissions in patients with atrial fibrillation receiving antithrombotic therapy: results from the Tasmanian Atrial Fibrillation (TAF) study. *Eur J Clin Pharmacol*. 2017;73(12):1681-1689. doi:10.1007/s00228-017-2337-9

2. Alharbi FF, Souverein PC, de Groot MCH, et al. The impact of serum potassium-influencing antihypertensive drugs on the risk of out-of-hospital cardiac arrest: A case-control study. *Br J Clin Pharmacol*. 2017;83(11):2541-2548. doi:10.1111/bcp.13356

3. Amer KA, Hurren JR, Edwin SB, Cohen G. Regadenoson versus Dipyridamole: A Comparison of the Frequency of Adverse Events in Patients Undergoing Myocardial Perfusion Imaging. *Pharmacother J Hum Pharmacol Drug Ther*. 2017;37(6):657-661. doi:10.1002/phar.1940

4. Bakhriansyah M, Souverein PC, de Boer A, Klungel OH. Gastrointestinal toxicity among patients taking selective COX-2 inhibitors or conventional NSAIDs, alone or combined with proton pump inhibitors: a case-control study. *Pharmacoepidemiol Drug Saf*. 2017;26(10):1141-1148. doi:10.1002/pds.4183

5. Bali V, Chatterjee S, Johnson ML, Chen H, Carnahan RM, Aparasu RR. Risk of Mortality in Elderly Nursing Home Patients with Depression Using Paroxetine. *Pharmacother J Hum Pharmacol Drug Ther*. 2017;37(3):287-296. doi:10.1002/phar.1898

6. Bally M, Beauchamp M-E, Abrahamowicz M, Nadeau L, Brophy JM. Risk of acute myocardial infarction with real-world NSAIDs depends on dose and timing of exposure. *Pharmacoepidemiol Drug Saf*. 2018;27(1):69-77. doi:10.1002/pds.4358

7. Bérard A, Sheehy O, Zhao J-P, Vinet É, Bernatsky S, Abrahamowicz M. SSRI and SNRI use during pregnancy and the risk of persistent pulmonary hypertension of the newborn. *Br J Clin Pharmacol*. 2017;83(5):1126-1133. doi:10.1111/bcp.13194

8. Bezin J, Groenwold RHH, Ali MS, et al. Comparative effectiveness of recommended versus less intensive drug combinations in secondary prevention of acute coronary syndrome. *Pharmacoepidemiol Drug Saf*. 2017;26(3):285-293. doi:10.1002/pds.4171

9. Biffi A, Rea F, Scotti L, et al. Antidepressants and the risk of arrhythmia in elderly affected by a previous cardiovascular disease: a real-life investigation from Italy. *Eur J Clin Pharmacol*. 2018;74(1):119-129. doi:10.1007/s00228-017-2352-x

10. Brassard P, Wu JW, Ernst P, Dell’Aniello S, Smiechowski B, Suissa S. The effect of statins on influenza-like illness morbidity and mortality. *Pharmacoepidemiol Drug Saf*. 2017;26(1):63-70. doi:10.1002/pds.4112

11. Busby J, Murray L, Mills K, Zhang S-D, Liberante F, Cardwell CR. A combined connectivity mapping and pharmacoepidemiology approach to identify existing medications with breast cancer causing or preventing properties. *Pharmacoepidemiol Drug Saf*. 2018;27(1):78-86. doi:10.1002/pds.4345

12. Cantarutti A, Merlino L, Giaquinto C, Corrao G. Use of antidepressant medication in pregnancy and adverse neonatal outcomes: A population-based investigation. *Pharmacoepidemiol Drug Saf*. 2017;26(9):1100-1108. doi:10.1002/pds.4242

13. Carman WJ, Accortt NA, Anthony MS, Iles J, Enger C. Pregnancy and infant outcomes including major congenital malformations among women with chronic inflammatory arthritis or psoriasis, with and without etanercept use. *Pharmacoepidemiol Drug Saf*. 2017;26(9):1109-1118. doi:10.1002/pds.4261

14. Castelpietra G, Gobbato M, Valent F, De Vido C, Balestrieri M, Isacsson G. Antidepressant use in suicides: a case-control study from the Friuli Venezia Giulia Region, Italy, 2005–2014. *Eur J Clin Pharmacol*. 2017;73(7):883-890. doi:10.1007/s00228-017-2236-0

15. Cieri NE, Kusmierski K, Lackie C, Van Opdorp A, Hassan AK. Retrospective Evaluation of Postoperative Adverse Drug Events in Patients Receiving Rivaroxaban After Major Orthopedic Surgery Compared with Standard Therapy in a Community Hospital. *Pharmacother J Hum Pharmacol Drug Ther*. 2017;37(2):170-176. doi:10.1002/phar.1888

16. Coons JC, Iasella CJ, Chanas T, et al. Comparative Effectiveness and Safety Analysis of Dual Antiplatelet Therapies Within an Integrated Delivery System. *Ann Pharmacother*. 2017;51(8):649-655. doi:10.1177/1060028017706977

17. EuroCoord TEP and PHCC (EPPICC) study group in. Safety of zidovudine/lamivudine scored tablets in children with HIV infection in Europe and Thailand. *Eur J Clin Pharmacol*. 2017;73(4):463-468. doi:10.1007/s00228-016-2182-2

18. Fang G, Annis IE, Farley JF, et al. Incidence of and Risk Factors for Severe Adverse Events in Elderly Patients Taking Angiotensin-Converting Enzyme Inhibitors or Angiotensin II Receptor Blockers after an Acute Myocardial Infarction. *Pharmacother J Hum Pharmacol Drug Ther*. 2018;38(1):29-41. doi:10.1002/phar.2051

19. Ferrajolo C, Verhamme KMC, Trifirò G, et al. Antibiotic-Induced Liver Injury in Paediatric Outpatients: A Case-Control Study in Primary Care Databases. *Drug Saf*. 2017;40(4):305-315. doi:10.1007/s40264-016-0493-y

20. Gagne JJ, Houstoun M, Reichman ME, Hampp C, Marshall JH, Toh S. Safety assessment of niacin in the US Food and Drug Administration’s mini-sentinel system. *Pharmacoepidemiol Drug Saf*. 2018;27(1):30-37. doi:10.1002/pds.4343

21. Gieling EM, van den Ham HA, van Onzenoort H, et al. Risk of major bleeding and stroke associated with the use of vitamin K antagonists, nonvitamin K antagonist oral anticoagulants and aspirin in patients with atrial fibrillation: a cohort study. *Br J Clin Pharmacol*. 2017;83(8):1844-1859. doi:10.1111/bcp.13265

22. Giner-Soriano M, Roso-Llorach A, Vedia Urgell C, et al. Effectiveness and safety of drugs used for stroke prevention in a cohort of non-valvular atrial fibrillation patients from a primary care electronic database. *Pharmacoepidemiol Drug Saf*. 2017;26(1):97-107. doi:10.1002/pds.4137

23. González-Pérez A, Sáez ME, Johansson S, Nagy P, García Rodríguez LA. Mortality in patients who discontinue low-dose acetylsalicylic acid therapy after upper gastrointestinal bleeding. *Pharmacoepidemiol Drug Saf*. 2017;26(2):215-222. doi:10.1002/pds.4140

24. Gorricho J, Garjón J, Alonso A, et al. Use of oral antidiabetic agents and risk of community-acquired pneumonia: a nested case-control study. *Br J Clin Pharmacol*. 2017;83(9):2034-2044. doi:10.1111/bcp.13288

25. Grabarczyk TR. Observational Comparative Effectiveness of Pharmaceutical Treatments for Obesity within the Veterans Health Administration. *Pharmacother J Hum Pharmacol Drug Ther*. 2018;38(1):19-28. doi:10.1002/phar.2048

26. Grelle JL, Kutter SN, Giruzzi ME, Tawwater JC. Impact of Insulin Detemir Administration Time on Hypoglycemia Rates in Hospitalized Patients. *Pharmacother J Hum Pharmacol Drug Ther*. 2017;37(12):1523-1529. doi:10.1002/phar.2045

27. Hagberg KW, Divan HA, Nickel JC, Jick SS. Risk of Incident Antidepressant-Treated Depression Associated with Use of 5α-Reductase Inhibitors Compared with Use of α-Blockers in Men with Benign Prostatic Hyperplasia: A Population-Based Study Using the Clinical Practice Research Datalink. *Pharmacother J Hum Pharmacol Drug Ther*. 2017;37(5):517-527. doi:10.1002/phar.1925

28. Ham AC, van Dijk SC, Swart KMA, et al. Beta-blocker use and fall risk in older individuals: Original results from two studies with meta-analysis. *Br J Clin Pharmacol*. 2017;83(10):2292-2302. doi:10.1111/bcp.13328

29. Hedna K, Andersson Sundell K, Hamidi A, Skoog I, Gustavsson S, Waern M. Antidepressants and suicidal behaviour in late life: a prospective population-based study of use patterns in new users aged 75 and above. *Eur J Clin Pharmacol*. 2018;74(2):201-208. doi:10.1007/s00228-017-2360-x

30. Ho JM-W, Macdonald EM, Luo J, et al. Pregabalin and heart failure: A population-based study. *Pharmacoepidemiol Drug Saf*. 2017;26(9):1087-1092. doi:10.1002/pds.4239

31. Hu X, Cheng J, Li C. Effects of rosuvastatin and atorvastatin on nonsustained ventricular tachycardia in patients with ST-elevation myocardial infarction: a retrospective analysis. *Eur J Clin Pharmacol*. 2018;74(1):29-35. doi:10.1007/s00228-017-2338-8

32. Jhang K-M, Huang J-Y, Nfor ON, et al. Extrapyramidal symptoms after exposure to calcium channel blocker-flunarizine or cinnarizine. *Eur J Clin Pharmacol*. 2017;73(7):911-916. doi:10.1007/s00228-017-2247-x

33. Jobski K, Kollhorst B, Garbe E, Schink T. The Risk of Ischemic Cardio- and Cerebrovascular Events Associated with Oxycodone–Naloxone and Other Extended-Release High-Potency Opioids: A Nested Case–Control Study. *Drug Saf*. 2017;40(6):505-515. doi:10.1007/s40264-017-0511-8

34. Jorgenson MR, Descourouez JL, Leverson GE, et al. High-Dose Acyclovir for Cytomegalovirus Prophylaxis in Seropositive Abdominal Transplant Recipients. *Ann Pharmacother*. 2018;52(1):5-10. doi:10.1177/1060028017728296

35. Jorgenson MR, Descourouez JL, Singh T, Astor BC, Panzer SE. Malignancy in Renal Transplant Recipients Exposed to Cyclophosphamide Prior to Transplantation for the Treatment of Native Glomerular Disease. *Pharmacother J Hum Pharmacol Drug Ther*. 2018;38(1):51-57. doi:10.1002/phar.2059

36. Kaye JA, Margulis A V., Fortuny J, et al. Cancer Incidence after Initiation of Antimuscarinic Medications for Overactive Bladder in the United Kingdom: Evidence for Protopathic Bias. *Pharmacother J Hum Pharmacol Drug Ther*. 2017;37(6):673-683. doi:10.1002/phar.1932

37. Kim K, Lee TA, Ardati AK, DiDomenico RJ, Touchette DR, Walton SM. Comparative Effectiveness of Oral Antiplatelet Agents in Patients with Acute Coronary Syndrome. *Pharmacother J Hum Pharmacol Drug Ther*. 2017;37(8):877-887. doi:10.1002/phar.1961

38. Kim TM, Kim H, Jeong YJ, et al. The differences in the incidence of diabetes mellitus and prediabetes according to the type of HMG-CoA reductase inhibitors prescribed in Korean patients. *Pharmacoepidemiol Drug Saf*. 2017;26(10):1156-1163. doi:10.1002/pds.4237

39. King JB, Shah RU, Sainski-Nguyen A, Biskupiak J, Munger MA, Bress AP. Effect of Inpatient Dobutamine versus Milrinone on Out-of-Hospital Mortality in Patients with Acute Decompensated Heart Failure. *Pharmacother J Hum Pharmacol Drug Ther*. 2017;37(6):662-672. doi:10.1002/phar.1939

40. Kreys ED, Frei CR, Villarreal SM, Bollinger MJ, Jones X, Koeller JM. Evaluation of Long-Term Chronic Myeloid Leukemia Treatment Practices with Tyrosine Kinase Inhibitors in a National Cohort of Veterans. *Pharmacother J Hum Pharmacol Drug Ther*. 2017;37(3):278-286. doi:10.1002/phar.1893

41. Kuoppala J, Enlund H, Pulkkinen J, et al. ACE inhibitors and the risk of acute pancreatitis-a population-based case-control study. *Pharmacoepidemiol Drug Saf*. 2017;26(7):853-857. doi:10.1002/pds.4179

42. Lai C-H, Yang Y-H, Chen P-C, King Y-C, Liu C-Y. Retinal vascular complications associated with interferon-ribavirin therapy for chronic hepatitis C: A population-based study. *Pharmacoepidemiol Drug Saf*. 2018;27(2):191-198. doi:10.1002/pds.4363

43. Larivée N, Suissa S, Coulombe J, Tagalakis V, Filion KB. Drospirenone-Containing Oral Contraceptive Pills and the Risk of Venous Thromboembolism: An Assessment of Risk in First-Time Users and Restarters. *Drug Saf*. 2017;40(7):583-596. doi:10.1007/s40264-017-0525-2

44. LaRue HA, Peksa GD, Shah SC. A Comparison of Insulin Doses for the Treatment of Hyperkalemia in Patients with Renal Insufficiency. *Pharmacother J Hum Pharmacol Drug Ther*. 2017;37(12):1516-1522. doi:10.1002/phar.2038

45. Lehto M, Niiranen J, Korhonen P, et al. Quality of warfarin therapy and risk of stroke, bleeding, and mortality among patients with atrial fibrillation: results from the nationwide FinWAF Registry. *Pharmacoepidemiol Drug Saf*. 2017;26(6):657-665. doi:10.1002/pds.4194

46. Leonard CE, Han X, Brensinger CM, et al. Comparative risk of serious hypoglycemia with oral antidiabetic monotherapy: A retrospective cohort study. *Pharmacoepidemiol Drug Saf*. 2018;27(1):9-18. doi:10.1002/pds.4337

47. Lin H-F, Liao K-F, Chang C-M, Lin C-L, Lai S-W. Tamoxifen usage correlates with increased risk of Parkinson’s disease in older women with breast cancer: a case–control study in Taiwan. *Eur J Clin Pharmacol*. 2018;74(1):99-107. doi:10.1007/s00228-017-2341-0

48. Lin H-F, Liao K-F, Chang C-M, Lin C-L, Lai S-W. Association of use of selective serotonin reuptake inhibitors with risk of acute pancreatitis: a case-control study in Taiwan. *Eur J Clin Pharmacol*. 2017;73(12):1615-1621. doi:10.1007/s00228-017-2328-x

49. Lin H-F, Liao K-F, Chang C-M, Lin C-L, Lai S-W. Correlation between proton pump inhibitors and risk of pyogenic liver abscess. *Eur J Clin Pharmacol*. 2017;73(8):1019-1025. doi:10.1007/s00228-017-2256-9

50. Lin H-L, Lin H-C, Tseng Y-F, Chen S-C, Hsu C-Y. Risk of parkinsonism induced by flunarizine or cinnarizine: a population-based study. *Eur J Clin Pharmacol*. 2017;73(3):365-371. doi:10.1007/s00228-016-2181-3

51. Lo Re V, Zeldow B, Kallan MJ, et al. Risk of liver decompensation with cumulative use of mitochondrial toxic nucleoside analogues in HIV/hepatitis C virus coinfection. *Pharmacoepidemiol Drug Saf*. 2017;26(10):1172-1181. doi:10.1002/pds.4258

52. Mackenzie IS, Morant S V., Wei L, Thompson AM, MacDonald TM. Spironolactone use and risk of incident cancers: a retrospective, matched cohort study. *Br J Clin Pharmacol*. 2017;83(3):653-663. doi:10.1111/bcp.13152

53. Margulis A V., Hallas J, Pottegård A, et al. Comparison of cardiovascular events among treatments for overactive bladder: a Danish nationwide cohort study. *Eur J Clin Pharmacol*. 2018;74(2):193-199. doi:10.1007/s00228-017-2359-3

54. McGrogan A, Snowball J, Charlton RA. Statins during pregnancy: a cohort study using the General Practice Research Database to investigate pregnancy loss. *Pharmacoepidemiol Drug Saf*. 2017;26(7):843-852. doi:10.1002/pds.4176

55. Meid AD, von Medem A, Heider D, et al. Investigating the Additive Interaction of QT-Prolonging Drugs in Older People Using Claims Data. *Drug Saf*. 2017;40(2):133-144. doi:10.1007/s40264-016-0477-y

56. Momen NC, Munk-Olsen T, Li J, et al. Antidepressant use during pregnancy and childhood cancer in the offspring. *Pharmacoepidemiol Drug Saf*. 2018;27(1):114-118. doi:10.1002/pds.4352

57. Mori N, Kamimura Y, Kimura Y, Hirose S, Aoki Y, Bito S. Comparative analysis of lactic acidosis induced by linezolid and vancomycin therapy using cohort and case–control studies of incidence and associated risk factors. *Eur J Clin Pharmacol*. 2018;74(4):405-411. doi:10.1007/s00228-017-2377-1

58. Mousavi M, Zapolskaya T, Scipione MR, Louie E, Papadopoulos J, Dubrovskaya Y. Comparison of Rates of Nephrotoxicity Associated with Vancomycin in Combination with Piperacillin-Tazobactam Administered as an Extended versus Standard Infusion. *Pharmacother J Hum Pharmacol Drug Ther*. 2017;37(3):379-385. doi:10.1002/phar.1901

59. Muanda FT, Sheehy O, Bérard A. Use of antibiotics during pregnancy and the risk of major congenital malformations: a population based cohort study. *Br J Clin Pharmacol*. 2017;83(11):2557-2571. doi:10.1111/bcp.13364

60. Nardone B, Majewski S, Kim AS, et al. Melanoma and Non-Melanoma Skin Cancer Associated with Angiotensin-Converting-Enzyme Inhibitors, Angiotensin-Receptor Blockers and Thiazides: A Matched Cohort Study. *Drug Saf*. 2017;40(3):249-255. doi:10.1007/s40264-016-0487-9

61. Nishtala PS, Chyou T. Real-world risk of diabetes with antipsychotic use in older New Zealanders: a case-crossover study. *Eur J Clin Pharmacol*. 2017;73(2):233-239. doi:10.1007/s00228-016-2158-2

62. Noize P, Grelaud A, Bay J-O, et al. Real-life patterns of use, safety and effectiveness of sunitinib in first-line therapy of metastatic renal cell carcinoma: the SANTORIN cohort study. *Pharmacoepidemiol Drug Saf*. 2017;26(12):1561-1569. doi:10.1002/pds.4228

63. Ou H-T, Chang K-C, Li C-Y, Wu J-S. Comparative cardiovascular risks of dipeptidyl peptidase 4 inhibitors with other second- and third-line antidiabetic drugs in patients with type 2 diabetes. *Br J Clin Pharmacol*. 2017;83(7):1556-1570. doi:10.1111/bcp.13241

64. Ou H-T, Lee T-Y, Du Y-F, Li C-Y. Comparative risks of diabetes-related complications of basal insulins: a longitudinal population-based cohort of type 1 diabetes 1999-2013 in Taiwan. *Br J Clin Pharmacol*. 2018;84(2):379-391. doi:10.1111/bcp.13461

65. Perreault S, de Denus S, White M, et al. Older adults with heart failure treated with carvedilol, bisoprolol, or metoprolol tartrate: risk of mortality. *Pharmacoepidemiol Drug Saf*. 2017;26(1):81-90. doi:10.1002/pds.4132

66. Poluzzi E, Diemberger I, De Ridder M, et al. Use of antihistamines and risk of ventricular tachyarrhythmia: a nested case-control study in five European countries from the ARITMO project. *Eur J Clin Pharmacol*. 2017;73(11):1499-1510. doi:10.1007/s00228-017-2317-0

67. Qian CJ, Coulombe J, Suissa S, Ernst P. Pneumonia risk in asthma patients using inhaled corticosteroids: a quasi-cohort study. *Br J Clin Pharmacol*. 2017;83(9):2077-2086. doi:10.1111/bcp.13295

68. Quinn KL, Macdonald EM, Gomes T, et al. Macrolides, Digoxin Toxicity and the Risk of Sudden Death: A Population-Based Study. *Drug Saf*. 2017;40(9):835-840. doi:10.1007/s40264-017-0539-9

69. Quinn KL, Macdonald EM, Mamdani MM, Diong C, Juurlink DN, (CDSERN) for the CDS and ERN. Lipophilic Statins and the Risk of Intracranial Hemorrhage Following Ischemic Stroke: A Population-Based Study. *Drug Saf*. 2017;40(10):887-893. doi:10.1007/s40264-017-0552-z

70. Rea F, Bonassi S, Vitale C, et al. Exposure to statins is associated to fracture risk reduction in elderly people with cardiovascular disease: evidence from the AIFA-I-GrADE observational project. *Pharmacoepidemiol Drug Saf*. 2017;26(7):775-784. doi:10.1002/pds.4206

71. Reichman ME, Wernecke M, Graham DJ, et al. Antihypertensive drug associated angioedema: effect modification by race/ethnicity. *Pharmacoepidemiol Drug Saf*. 2017;26(10):1190-1196. doi:10.1002/pds.4260

72. Russo-Alvarez G, Martinez KA, Valente M, et al. Thromboembolic and Major Bleeding Events With Rivaroxaban Versus Warfarin Use in a Real-World Setting. *Ann Pharmacother*. 2018;52(1):19-25. doi:10.1177/1060028017727290

73. Rutter WC, Burgess DS. Acute Kidney Injury in Patients Treated with IV Beta-Lactam/Beta-Lactamase Inhibitor Combinations. *Pharmacother J Hum Pharmacol Drug Ther*. 2017;37(5):593-598. doi:10.1002/phar.1918

74. Samp JC, Joo MJ, Schumock GT, Calip GS, Pickard AS, Lee TA. Risk of Cardiovascular and Cerebrovascular Events in COPD Patients Treated With Long-Acting β _2_ -Agonist Combined With a Long-Acting Muscarinic or Inhaled Corticosteroid. *Ann Pharmacother*. 2017;51(11):945-953. doi:10.1177/1060028017719716

75. Samp JC, Joo MJ, Schumock GT, Calip GS, Pickard AS, Lee TA. Comparative Effectiveness of Long-Acting Beta _2_ -Agonist Combined with a Long-Acting Muscarinic Antagonist or Inhaled Corticosteroid in Chronic Obstructive Pulmonary Disease. *Pharmacother J Hum Pharmacol Drug Ther*. 2017;37(4):447-455. doi:10.1002/phar.1913

76. Samuel NG, Seifert CF. Risk of Bleeding in Patients on Full-Dose Enoxaparin With Venous Thromboembolism and Selective Serotonin Reuptake Inhibitors. *Ann Pharmacother*. 2017;51(3):226-231. doi:10.1177/1060028016677309

77. Shalev V, Sharman Moser S, Goldshtein I, et al. Adherence With Bisphosphonates and Long-Term Risk of Hip Fractures: A Nested Case-Control Study Using Real-World Data. *Ann Pharmacother*. 2017;51(9):757-767. doi:10.1177/1060028017710482

78. Shin J-Y, Eberg M, Ernst P, Filion KB. Statin potency and the risk of hospitalization for community-acquired pneumonia. *Br J Clin Pharmacol*. 2017;83(6):1319-1327. doi:10.1111/bcp.13208

79. Shlomo M, Gorodischer R, Daniel S, et al. The Fetal Safety of Enoxaparin Use During Pregnancy: A Population-Based Retrospective Cohort Study. *Drug Saf*. 2017;40(11):1147-1155. doi:10.1007/s40264-017-0573-7

80. Simard P, Presse N, Roy L, et al. Association Between Metformin Adherence and All-Cause Mortality Among New Users of Metformin: A Nested Case-Control Study. *Ann Pharmacother*. 2018;52(4):305-313. doi:10.1177/1060028017743517

81. Stolk LM, de Vries F, Ebbelaar C, et al. Risk of myocardial infarction in patients with atrial fibrillation using vitamin K antagonists, aspirin or direct acting oral anticoagulants. *Br J Clin Pharmacol*. 2017;83(8):1835-1843. doi:10.1111/bcp.13264

82. Su X, Yuan W, Chen J, et al. Prenatal exposure to β2-adrenoreceptor agonists and the risk of autism spectrum disorders in offspring. *Pharmacoepidemiol Drug Saf*. 2017;26(7):812-818. doi:10.1002/pds.4214

83. Svendsen T de K, Nørregaard Hansen P, García Rodríguez LA, et al. Statins and polyneuropathy revisited: case-control study in Denmark, 1999-2013. *Br J Clin Pharmacol*. 2017;83(9):2087-2095. doi:10.1111/bcp.13298

84. Thygesen LC, Pottegård A, Ersbøll AK, Friis S, Stürmer T, Hallas J. External adjustment of unmeasured confounders in a case-control study of benzodiazepine use and cancer risk. *Br J Clin Pharmacol*. 2017;83(11):2517-2527. doi:10.1111/bcp.13342

85. Tran Y-H, Groen H, Bergman JEH, Hak E, Wilffert B. Exposure to reactive intermediate-inducing drugs during pregnancy and the incident use of psychotropic medications among children. *Pharmacoepidemiol Drug Saf*. 2017;26(3):265-273. doi:10.1002/pds.4161

86. Valent F. New oral anticoagulant prescription rate and risk of bleeding in an Italian region. *Pharmacoepidemiol Drug Saf*. 2017;26(10):1205-1212. doi:10.1002/pds.4279

87. Vozoris NT, Wang X, Austin PC, et al. Adverse cardiac events associated with incident opioid drug use among older adults with COPD. *Eur J Clin Pharmacol*. 2017;73(10):1287-1295. doi:10.1007/s00228-017-2278-3

88. Vozoris NT, Wang X, Austin PC, et al. Incident diuretic drug use and adverse respiratory events among older adults with chronic obstructive pulmonary disease. *Br J Clin Pharmacol*. 2018;84(3):579-589. doi:10.1111/bcp.13465

89. Wändell P, Carlsson AC, Holzmann M, et al. Association between antithrombotic treatment and hemorrhagic stroke in patients with atrial fibrillation—a cohort study in primary care. *Eur J Clin Pharmacol*. 2017;73(2):215-221. doi:10.1007/s00228-016-2152-8

90. Wang C-Y, Lin Z-F, Lee C-M, et al. Concomitant use of calcium channel blockers with dual antiplatelet therapy and re-hospitalization for acute coronary syndrome. *Pharmacoepidemiol Drug Saf*. 2017;26(3):229-238. doi:10.1002/pds.4147

91. Wei L, Ratnayake L, Phillips G, et al. Acid-suppression medications and bacterial gastroenteritis: a population-based cohort study. *Br J Clin Pharmacol*. 2017;83(6):1298-1308. doi:10.1111/bcp.13205

**S3 – Results from stratified analyses**

**Table S1 - Reporting quality of the studies included in this systematic review of the quality of reporting in pharmacoepidemiology stratified per exposure definition. For each specific item, the number of studies reporting that item is shown.**

|  | *Intention to treat (n=24) ^†^* | | *>= 1 prescription during a certain period (n=19) ^†^* | | *Time-varying exposure (n=43) ^†^* | |
| --- | --- | --- | --- | --- | --- | --- |
|  | Studies, *n ^§^* | Reported, n *(%)* | Studies, *n ^§^* | Reported, n *(%)* | Studies, *n ^§^* | Reported, n *(%)* |
| **1 Type of exposure** | 24 | 24 (100) | 19 | 19 (100) | 43 | 43 (100) |
| **2 Exposure risk window (ERW)** | 24 | 17 (71) | 19 | 16 (84) | 43 | 39 (91) |
| **3 Induction period ^‡^**   - *Explicit* - *Implicit* | 24 | 20 (83)  *5 (21)*  *15 (63)* | 19 | 17 (89)  *2 (11)*  *15 (79)* | 43 | 39 (91)  *6 (14)*  *33 (77)* |
| **4 Stockpiling** | 0 | *NA* | 0 | *NA* | 42 | 3 (7) |
| **5 Bridging exposure episodes** | 0 | *NA* | 0 | *NA* | 42 | 18 (43) |
| **6 Exposure extension ^‡^**   - *Explicit* - *Implicit* | 0 | *NA* | 3 | 2 (67)  *1 (33)*  *1 (33)* | 43 | 24 (56)  *16 (37)*  *8 (19)* |
| **7 Switching/ add on** | 21 | 12 (57) | 14 | 7 (50) | 42 | 29 (69) |
| **8 Codes** | 24 | 5 (21) | 19 | 8 (42) | 43 | 10 (23) |
| **9 Frequency and temporality of codes** | 24 | 18 (75) | 19 | 16 (84) | 43 | 38 (88) |
| **10 Care setting** | 24 | 18 (75) | 19 | 13 (68) | 43 | 33 (77) |
| **11 Exposure Assessment Window (EAW)** | 24 | 23 (96) | 19 | 18 (95) | 43 | 43 (100) |

^†^ The exposure definition was divided in five categories: 1) intention to treat: exposure at baseline is included as a time-fixed variable in the model; 2) the presence of ≥1 prescriptions during a certain time period, for example during pregnancy or during the last 12 months prior to the event; 3) time-varying: episodes of (non)exposure are constructed based on duration of each prescription; and 4) other, including measures of adherence and (cumulative) dose and cumulative dose.
^‡^ When explicitly mentioning an introduction period, a period after the index date is clearly excluded in the exposure risk window. Stating that the follow-up started on the day of the first prescription implies implicitly that there was no induction period. The same reasoning also applies to the extension period.
^§^ Total number of studies for which this item was applicable.

**Table S2 - Reporting quality of the studies included in this systematic review of the quality of reporting in pharmacoepidemiology stratified per type of study design. For each specific item, the number of studies reporting that item is shown.**

|  | Cohort (n=64) | | Case-control or case-crossover (n=29) | |
| --- | --- | --- | --- | --- |
|  | Studies, *n* ^‡^ | Reported, n *(%)* | Studies, *n* ^‡^ | Reported, n *(%)* |
| 1 Type of exposure | 64 | 62 (95) | 29 | 29 (100) |
| 2 Exposure risk window (ERW) | 64 | 51 (80) | 29 | 27 (93) |
| 3 Induction period ^†^   - *Explicit* - *Implicit* | 63 | 55 (87)  *9 (14)*  *46 (73)* | 29 | 28 (97)  *5 (17)*  *23 (79)* |
| 4 Stockpiling | 30 | 1 (3) | 18 | 4 (22) |
| 5 Bridging exposure episodes | 30 | 12 (40) | 15 | 6 (40) |
| 6 Exposure extension ^†^   - *Explicit* - *Implicit* | 34 | 14 (41)  *6 (18)*  *8 (24)* | 16 | 13 (81)  *11 (69)*  *2 (13)* |
| 7 Switching/ add on | 55 | 34 (62) | 28 | 17 (61) |
| 8 Codes | 64 | 12 (19) | 29 | 13 (45) |
| 9 Frequency and temporality of codes | 64 | 51 (80) | 29 | 27 (93) |
| 10 Care setting | 64 | 48 (75) | 29 | 21 (72) |
| 11 Exposure Assessment Window (EAW) | 64 | 63 (98) | 29 | 28 (97) |

^†^ When explicitly mentioning an introduction period, a period after the index date is clearly excluded in the exposure risk window. Stating that the follow-up started on the day of the first prescription implies implicitly that there was no induction period. The same reasoning also applies to the extension period.

^‡^ Total number of studies for which this item was applicable.

**Table S3 - Reporting quality of the studies included in this systematic review of the quality of reporting in pharmacoepidemiology stratified per number of included subjects. For each specific item, the number of studies reporting that item is shown.**

|  | 250 – 1,000 patients (n=13) | | 1,001 – 10,000 patients (n=30) | | 10,001 – 100,000 patients (n=24) | | >100,000 patients (n=24) | |
| --- | --- | --- | --- | --- | --- | --- | --- | --- |
|  | Studies, *n* ^‡^ | Reported, n *(%)* | Studies, *n* ^‡^ | Reported, n *(%)* | Studies, *n* ^‡^ | Reported, n *(%)* | Studies, *n* ^‡^ | Reported, n *(%)* |
| 1 Type of exposure | 13 | 11 (85) | 30 | 30 (100) | 24 | 24 (100) | 24 | 24 (100) |
| 2 Exposure risk window (ERW) | 13 | 7 (54) | 30 | 27 (90) | 24 | 22 (92) | 24 | 21 (88) |
| 3 Induction period ^†^   - *Explicit* - *Implicit* | 12 | 10 (83)  *0 (0)*  *10 (83)* | 30 | 27 (90)  *6 (20)*  *21 (70)* | 24 | 22 (92)  *3 (13)*  *19 (79)* | 23 | 22 (96)  *5 (22)*  *17 (74)* |
| 4 Stockpiling | 6 | 0 (0) | 16 | 3 (19) | 14 | 1 (7) | 11 | 1 (9) |
| 5 Bridging exposure episodes | 6 | 0 (0) | 14 | 5 (36) | 13 | 4 (31) | 11 | 9 (82) |
| 6 Exposure extension ^†^   - *Explicit* - *Implicit* | 8 | 2 (25)  *1 (13)*  *1 (13)* | 16 | 9 (56)  *5 (31)*  *4 (25)* | 14 | 10 (71)  *6 (43)*  *4 (29)* | 11 | 6 (55)  *5 (45)*  *1 (9)* |
| 7 Switching/ add on | 12 | 5 (42) | 25 | 14 (56) | 23 | 15 (65) | 21 | 16 (76) |
| 8 Codes | 13 | 0 (0) | 30 | 11 (37) | 24 | 6 (25) | 24 | 7 (29) |
| 9 Frequency and temporality of codes | 13 | 6 (46) | 30 | 27 (90) | 24 | 21 (88) | 24 | 23 (96) |
| 10 Care setting | 13 | 12 (92) | 30 | 21 (70) | 24 | 16 (67) | 24 | 18 (75) |
| 11 Exposure Assessment Window (EAW) | 13 | 13 (100) | 30 | 28 (93) | 24 | 24 (100) | 24 | 24 (100) |

^†^ When explicitly mentioning an introduction period, a period after the index date is clearly excluded in the exposure risk window. Stating that the follow-up started on the day of the first prescription implies implicitly that there was no induction period. The same reasoning also applies to the extension period.

^‡^ Total number of studies for which this item was applicable.

**Table S4 - Reporting quality of the studies included in this systematic review of the quality of reporting in pharmacoepidemiology stratified per type of outcome. For each specific item, the number of studies reporting that item is shown.**

|  | Beneficial effects (n=18) | | Adverse effects (n=67) | | Both (n=6) | |
| --- | --- | --- | --- | --- | --- | --- |
|  | Studies, *n* ^‡^ | Reported, n *(%)* | Studies, *n* ^‡^ | Reported, n *(%)* | Studies, *n* ^‡^ | Reported, n *(%)* |
| 1 Type of exposure | 18 | 17 (94) | 67 | 66 (99) | 6 | 6 (100) |
| 2 Exposure risk window (ERW) | 18 | 16 (89) | 67 | 55 (82) | 6 | 6 (100) |
| 3 Induction period ^†^   - *Explicit* - *Implicit* | 17 | 17 (100)  *6 (35)*  *11 (65)* | 67 | 58 (87)  *8 (12)*  *50 (75)* | 6 | 6 (100)  *0 (0)*  *6 (100)* |
| 4 Stockpiling | 10 | 3 (30) | 36 | 2 (6) | 1 | 0 (0) |
| 5 Bridging exposure episodes | 7 | 4 (57) | 36 | 14 (39) | 1 | 0 (0) |
| 6 Exposure extension ^†^   - *Explicit* - *Implicit* | 7 | 4 (57)  *1 (14)*  *3 (43)* | 40 | 22 (55)  *6 (15)*  *16 (40)* | 2 | 1 (50)  *0 (0)*  *1 (50)* |
| 7 Switching/ add on | 16 | 12 (75) | 60 | 35 (60) | 5 | 3 (60) |
| 8 Codes | 18 | 5 (28) | 67 | 18 (27) | 6 | 1 (17) |
| 9 Frequency and temporality of codes | 18 | 16 (89) | 67 | 55 (82) | 6 | 6 (100) |
| 10 Care setting | 18 | 13 (72) | 67 | 49 (73) | 6 | 5 (83) |
| 11 Exposure Assessment Window (EAW) | 18 | 18 (100) | 67 | 65 (97) | 6 | 6 (100) |

^†^ When explicitly mentioning an introduction period, a period after the index date is clearly excluded in the exposure risk window. Stating that the follow-up started on the day of the first prescription implies implicitly that there was no induction period. The same reasoning also applies to the extension period.

^‡^ Total number of studies for which this item was applicable.

**Table S5 - Reporting quality of the studies included in this systematic review of the quality of reporting in pharmacoepidemiology stratified per type of database. For each specific item, the number of studies reporting that item is shown.**

|  | Claims (n=41) | | Pharmacy (n=16) | | GP (n=17) | | Hospital (n=18) | |
| --- | --- | --- | --- | --- | --- | --- | --- | --- |
|  | Studies, *n* ^‡^ | Reported, n *(%)* | Studies, *n* ^‡^ | Reported, n *(%)* | Studies, *n* ^‡^ | Reported, n *(%)* | Studies, *n* ^‡^ | Reported, n *(%)* |
| 1 Type of exposure | 41 | 41 (100) | 16 | 16 (100) | 17 | 17 (100) | 18 | 18 (100) |
| 2 Exposure risk window (ERW) | 41 | 39 (95) | 16 | 15 (94) | 17 | 15 (88) | 18 | 9 (50) |
| 3 Induction period ^†^   - *Explicit* - *Implicit* | 41 | 39 (95)  *6 (15)*  *33 (80)* | 15 | 14 (93)  *5 (33)*  *9 (60)* | 17 | 15 (88)  *1 (6)*  *14 (82)* | 18 | 14 (78)  *2 (11)*  *12 (67)* |
| 4 Stockpiling | 20 | 5 (25) | 9 | 0 (0) | 14 | 0 (0) | 6 | 0 (0) |
| 5 Bridging exposure episodes | 17 | 12 (71) | 9 | 3 (33) | 14 | 3 (21) | 6 | 0 (0) |
| 6 Exposure extension ^†^   - *Explicit* - *Implicit* | 18 | 12 (67)  *5 (28)*  *7 (39)* | 9 | 5 (56)  *0 (0)*  *5 (56)* | 15 | 11 (73)  *4 (27)*  *7 (47)* | 9 | 2 (22)  *2 (22)*  *0 (0)* |
| 7 Switching/ add on | 37 | 27 (73) | 14 | 7 (50) | 16 | 10 (63) | 15 | 9 (60) |
| 8 Codes | 41 | 10 (24) | 16 | 11 (69) | 17 | 5 (29) | 18 | 0 (0) |
| 9 Frequency and temporality of codes | 41 | 40 (98) | 16 | 15 (94) | 17 | 15 (88) | 18 | 9 (50) |
| 10 Care setting | 41 | 22 (54) | 16 | 13 (81) | 17 | 17 (100) | 18 | 18 (100) |
| 11 Exposure Assessment Window (EAW) | 41 | 39 (95) | 16 | 16 (100) | 17 | 17 (100) | 18 | 18 (100) |

^†^ When explicitly mentioning an introduction period, a period after the index date is clearly excluded in the exposure risk window. Stating that the follow-up started on the day of the first prescription implies implicitly that there was no induction period. The same reasoning also applies to the extension period.

^‡^ Total number of studies for which this item was applicable.

**Table S6 - Reporting quality of the studies included in this systematic review of the quality of reporting in pharmacoepidemiology stratified per maximum of words allowed. For each specific item, the number of studies reporting that item is shown.**

|  | Short reports | | Original research articles | | | | | |
| --- | --- | --- | --- | --- | --- | --- | --- | --- |
|  | Word limit ≤1500 (n=4) | | Word limit 1500-3000 (n=32) ^§^ | | Word limit 3000-4000 (n=30) ^§^ | | Word limit ≥4000 (n=25) ^§^ | |
|  | Studies, *n* ^‡^ | Reported, n *(%)* | Studies, *n* ^‡^ | Reported, n *(%)* | Studies, *n* ^‡^ | Reported, n *(%)* | Studies, *n* ^‡^ | Reported, n *(%)* |
| 1 Type of exposure | 4 | 4 (100) | 32 | 32 (100) | 30 | 30 (100) | 25 | 25 (100) |
| 2 Exposure risk window (ERW) | 4 | 3 (75) | 32 | 30 (94) | 30 | 25 (83) | 25 | 19 (76) |
| 3 Induction period ^†^   - *Explicit* - *Implicit* | 4 | 3 (75)  *1 (25)*  *2 (50)* | 32 | 30 (94)  *6 (19)*  *24 (75)* | 30 | 26 (87)  *6 (20)*  *20 (67)* | 24 | 22 (92)  *20 (83)*  *2 (8)* |
| 4 Stockpiling | 1 | 0 (0) | 20 | 4 (20) | 13 | 0 (0) | 13 | 1 (7) |
| 5 Bridging exposure episodes | 1 | 0 (0) | 17 | 8 (47) | 13 | 6 (46) | 13 | 4 (31) |
| 6 Exposure extension ^†^   - *Explicit* - *Implicit* | 2 | 1 (50)  *0 (0)*  *1 (50)* | 18 | 10 (56)  *5 (28)*  *5 (28)* | 16 | 9 (56)  *6 (38)*  *3 (19)* | 13 | 7 (54)  *6 (46)*  *1 (7)* |
| 7 Switching/ add on | 4 | 3 (75) | 28 | 16 (57) | 27 | 17 (62) | 22 | 14 (64) |
| 8 Codes | 4 | 1 (25) | 32 | 8 (25) | 30 | 7 (23) | 25 | 8 (32) |
| 9 Frequency and temporality of codes | 4 | 3 (75) | 32 | 28 (88) | 30 | 25 (83) | 25 | 21 (84) |
| 10 Care setting | 4 | 2 (50) | 32 | 24 (75) | 30 | 24 (80) | 25 | 17 (68) |
| 11 Exposure Assessment Window (EAW) | 4 | 4 (100) | 32 | 31 (97) | 30 | 29 (97) | 25 | 24 (96) |

^†^ When explicitly mentioning an introduction period, a period after the index date is clearly excluded in the exposure risk window. Stating that the follow-up started on the day of the first prescription implies implicitly that there was no induction period. The same reasoning also applies to the extension period.

^‡^ Total number of studies for which this item was applicable.

^§^ Word limit per journal, according to the author guidelines of the journals: Annals of Pharmacotherapy 3000; British Journal of Clinical Pharmacology 3000-4000; Drug Safety 6000; European Journal of Clinical Pharmacology 8-10 pages of around 450 words (=4500 words maximum); Pharmacoepidemiology and Drug Safety 3500; and Pharmacotherapy 3500.

**Table S7 - Reporting quality of the studies included in this systematic review of the quality of reporting in pharmacoepidemiology stratified per route of administration (oral/inhaled vs IV/subcutaneous). For each specific item, the number of studies reporting that item is shown.**

|  | Oral/inhaled (n=80) | | IV/subcutaneous (n=11) | |
| --- | --- | --- | --- | --- |
|  | Studies, *n* ^‡^ | Reported, n *(%)* | Studies, *n* ^‡^ | Reported, n *(%)* |
| 1 Type of exposure | 80 | 80 (100) | 11 | 11 (100) |
| 2 Exposure risk window (ERW) | 80 | 71 (89) | 11 | 6 (55) |
| 3 Induction period ^†^   - *Explicit* - *Implicit* | 79 | 73 (92)  *11 (14)*  *62 (78)* | 11 | 8 (72)  *3 (27)*  *5 (45)* |
| 4 Stockpiling | 44 | 5 (11) | 3 | 0 (0) |
| 5 Bridging exposure episodes | 41 | 18 (44) | 3 | 0 (0) |
| 6 Exposure extension ^†^   - *Explicit* - *Implicit* | 43 | 26 (60)  *17 (40)*  *9 (21)* | 6 | 1 (17)  *0 (0)*  *1 (17)* |
| 7 Switching/ add on | 74 | 46 (62) | 7 | 4 (57) |
| 8 Codes | 80 | 23 (29) | 11 | 1 (9) |
| 9 Frequency and temporality of codes | 80 | 71 (89) | 11 | 6 (55) |
| 10 Care setting | 80 | 58 (73) | 11 | 9 (82) |
| 11 Exposure Assessment Window (EAW) | 80 | 78 (98) | 11 | 11 (100) |

^†^ When explicitly mentioning an introduction period, a period after the index date is clearly excluded in the exposure risk window. Stating that the follow-up started on the day of the first prescription implies implicitly that there was no induction period. The same reasoning also applies to the extension period.

^‡^ Total number of studies for which this item was applicable.

**S4 – interobserver agreement per item**

| Item | Scores | | | Kappa | Overall agreement * |
| --- | --- | --- | --- | --- | --- |
|  | *Yes* | *No* | *NA* |  |  |
| **1 Type of exposure** | 91 | 0 | 0 | 0,11 | 0,88 |
| **2 Exposure risk window (ERW)** | 77 | 14 | 0 | 0,49 | 0,82 |
| **3 Induction period** | 71 | 9 | 1 | 0,20 | 0,66 |
| **4 Stockpiling** | 5 | 42 | 33 | 0,33 | 0,64 |
| **5 Bridging exposure episodes** | 18 | 26 | 47 | 0,35 | 0,58 |
| **6 Exposure extension** | 27 | 22 | 42 | 0,28 | 0,52 |
| **7 Switching/ add on** | 50 | 31 | 10 | 0,33 | 0,61 |
| **8 Codes** | 24 | 67 | 0 | 0,75 | 0,90 |
| **9 Frequency and temporality of codes** | 77 | 14 | 0 | 0,35 | 0,76 |
| **10 Care setting** | 67 | 24 | 0 | 0,43 | 0,78 |
| **11 Exposure Assessment Window (EAW)** | 89 | 2 | 0 | 0,23 | 0,93 |

* calculated as the proportion of publications agreed upon directly.
