# Supplementary material for: Quality of reporting of drug exposure in pharmacoepidemiological studies
Source: Pharmacoepidemiol Drug Saf. 2020 May 11;29(9):1141–50. doi: 10.1002/pds.5020 (PMC7539966; doi:10.1002/pds.5020)
Supplement: Supplementary file 3 — Data S3. Supporting Information. [file PDS-29-1141-s003.docx]

# Supplementary materials - Results from stratified analyses (S3)

**Table S1 - Reporting quality of the studies included in this systematic review of the quality of reporting in pharmacoepidemiology stratified per exposure definition. For each specific item, the number of studies reporting that item is shown.**

|  | *Intention to treat (n=24) ^†^* | | *>= 1 prescription during a certain period (n=19) ^†^* | | *Time-varying exposure (n=43) ^†^* | |
| --- | --- | --- | --- | --- | --- | --- |
|  | Studies, *n ^§^* | Reported, n *(%)* | Studies, *n ^§^* | Reported, n *(%)* | Studies, *n ^§^* | Reported, n *(%)* |
| **1 Type of exposure** | 24 | 24 (100) | 19 | 19 (100) | 43 | 43 (100) |
| **2 Exposure risk window (ERW)** | 24 | 17 (71) | 19 | 16 (84) | 43 | 39 (91) |
| **3 Induction period ^‡^**   - *Explicit* - *Implicit* | 24 | 20 (83)  *5 (21)*  *15 (63)* | 19 | 17 (89)  *2 (11)*  *15 (79)* | 43 | 39 (91)  *6 (14)*  *33 (77)* |
| **4 Stockpiling** | 0 | *NA* | 0 | *NA* | 42 | 3 (7) |
| **5 Bridging exposure episodes** | 0 | *NA* | 0 | *NA* | 42 | 18 (43) |
| **6 Exposure extension ^‡^**   - *Explicit* - *Implicit* | 0 | *NA* | 3 | 2 (67)  *1 (33)*  *1 (33)* | 43 | 24 (56)  *16 (37)*  *8 (19)* |
| **7 Switching/ add on** | 21 | 12 (57) | 14 | 7 (50) | 42 | 29 (69) |
| **8 Codes** | 24 | 5 (21) | 19 | 8 (42) | 43 | 10 (23) |
| **9 Frequency and temporality of codes** | 24 | 18 (75) | 19 | 16 (84) | 43 | 38 (88) |
| **10 Care setting** | 24 | 18 (75) | 19 | 13 (68) | 43 | 33 (77) |
| **11 Exposure Assessment Window (EAW)** | 24 | 23 (96) | 19 | 18 (95) | 43 | 43 (100) |

^†^ The exposure definition was divided in five categories: 1) intention to treat: exposure at baseline is included as a time-fixed variable in the model; 2) the presence of ≥1 prescriptions during a certain time period, for example during pregnancy or during the last 12 months prior to the event; 3) time-varying: episodes of (non)exposure are constructed based on duration of each prescription; and 4) other, including measures of adherence and (cumulative) dose and cumulative dose.
^‡^ When explicitly mentioning an introduction period, a period after the index date is clearly excluded in the exposure risk window. Stating that the follow-up started on the day of the first prescription implies implicitly that there was no induction period. The same reasoning also applies to the extension period.
^§^ Total number of studies for which this item was applicable.

**Table S2 - Reporting quality of the studies included in this systematic review of the quality of reporting in pharmacoepidemiology stratified per type of study design. For each specific item, the number of studies reporting that item is shown.**

|  | Cohort (n=64) | | Case-control or case-crossover (n=29) | |
| --- | --- | --- | --- | --- |
|  | Studies, *n* ^‡^ | Reported, n *(%)* | Studies, *n* ^‡^ | Reported, n *(%)* |
| 1 Type of exposure | 64 | 62 (95) | 29 | 29 (100) |
| 2 Exposure risk window (ERW) | 64 | 51 (80) | 29 | 27 (93) |
| 3 Induction period ^†^   - *Explicit* - *Implicit* | 63 | 55 (87)  *9 (14)*  *46 (73)* | 29 | 28 (97)  *5 (17)*  *23 (79)* |
| 4 Stockpiling | 30 | 1 (3) | 18 | 4 (22) |
| 5 Bridging exposure episodes | 30 | 12 (40) | 15 | 6 (40) |
| 6 Exposure extension ^†^   - *Explicit* - *Implicit* | 34 | 14 (41)  *6 (18)*  *8 (24)* | 16 | 13 (81)  *11 (69)*  *2 (13)* |
| 7 Switching/ add on | 55 | 34 (62) | 28 | 17 (61) |
| 8 Codes | 64 | 12 (19) | 29 | 13 (45) |
| 9 Frequency and temporality of codes | 64 | 51 (80) | 29 | 27 (93) |
| 10 Care setting | 64 | 48 (75) | 29 | 21 (72) |
| 11 Exposure Assessment Window (EAW) | 64 | 63 (98) | 29 | 28 (97) |

^†^ When explicitly mentioning an introduction period, a period after the index date is clearly excluded in the exposure risk window. Stating that the follow-up started on the day of the first prescription implies implicitly that there was no induction period. The same reasoning also applies to the extension period.

^‡^ Total number of studies for which this item was applicable.

**Table S3 - Reporting quality of the studies included in this systematic review of the quality of reporting in pharmacoepidemiology stratified per number of included subjects. For each specific item, the number of studies reporting that item is shown.**

|  | 250 – 1,000 patients (n=13) | | 1,001 – 10,000 patients (n=30) | | 10,001 – 100,000 patients (n=24) | | >100,000 patients (n=24) | |
| --- | --- | --- | --- | --- | --- | --- | --- | --- |
|  | Studies, *n* ^‡^ | Reported, n *(%)* | Studies, *n* ^‡^ | Reported, n *(%)* | Studies, *n* ^‡^ | Reported, n *(%)* | Studies, *n* ^‡^ | Reported, n *(%)* |
| 1 Type of exposure | 13 | 11 (85) | 30 | 30 (100) | 24 | 24 (100) | 24 | 24 (100) |
| 2 Exposure risk window (ERW) | 13 | 7 (54) | 30 | 27 (90) | 24 | 22 (92) | 24 | 21 (88) |
| 3 Induction period ^†^   - *Explicit* - *Implicit* | 12 | 10 (83)  *0 (0)*  *10 (83)* | 30 | 27 (90)  *6 (20)*  *21 (70)* | 24 | 22 (92)  *3 (13)*  *19 (79)* | 23 | 22 (96)  *5 (22)*  *17 (74)* |
| 4 Stockpiling | 6 | 0 (0) | 16 | 3 (19) | 14 | 1 (7) | 11 | 1 (9) |
| 5 Bridging exposure episodes | 6 | 0 (0) | 14 | 5 (36) | 13 | 4 (31) | 11 | 9 (82) |
| 6 Exposure extension ^†^   - *Explicit* - *Implicit* | 8 | 2 (25)  *1 (13)*  *1 (13)* | 16 | 9 (56)  *5 (31)*  *4 (25)* | 14 | 10 (71)  *6 (43)*  *4 (29)* | 11 | 6 (55)  *5 (45)*  *1 (9)* |
| 7 Switching/ add on | 12 | 5 (42) | 25 | 14 (56) | 23 | 15 (65) | 21 | 16 (76) |
| 8 Codes | 13 | 0 (0) | 30 | 11 (37) | 24 | 6 (25) | 24 | 7 (29) |
| 9 Frequency and temporality of codes | 13 | 6 (46) | 30 | 27 (90) | 24 | 21 (88) | 24 | 23 (96) |
| 10 Care setting | 13 | 12 (92) | 30 | 21 (70) | 24 | 16 (67) | 24 | 18 (75) |
| 11 Exposure Assessment Window (EAW) | 13 | 13 (100) | 30 | 28 (93) | 24 | 24 (100) | 24 | 24 (100) |

^†^ When explicitly mentioning an introduction period, a period after the index date is clearly excluded in the exposure risk window. Stating that the follow-up started on the day of the first prescription implies implicitly that there was no induction period. The same reasoning also applies to the extension period.

^‡^ Total number of studies for which this item was applicable.

**Table S4 - Reporting quality of the studies included in this systematic review of the quality of reporting in pharmacoepidemiology stratified per type of outcome. For each specific item, the number of studies reporting that item is shown.**

|  | Beneficial effects (n=18) | | Adverse effects (n=67) | | Both (n=6) | |
| --- | --- | --- | --- | --- | --- | --- |
|  | Studies, *n* ^‡^ | Reported, n *(%)* | Studies, *n* ^‡^ | Reported, n *(%)* | Studies, *n* ^‡^ | Reported, n *(%)* |
| 1 Type of exposure | 18 | 17 (94) | 67 | 66 (99) | 6 | 6 (100) |
| 2 Exposure risk window (ERW) | 18 | 16 (89) | 67 | 55 (82) | 6 | 6 (100) |
| 3 Induction period ^†^   - *Explicit* - *Implicit* | 17 | 17 (100)  *6 (35)*  *11 (65)* | 67 | 58 (87)  *8 (12)*  *50 (75)* | 6 | 6 (100)  *0 (0)*  *6 (100)* |
| 4 Stockpiling | 10 | 3 (30) | 36 | 2 (6) | 1 | 0 (0) |
| 5 Bridging exposure episodes | 7 | 4 (57) | 36 | 14 (39) | 1 | 0 (0) |
| 6 Exposure extension ^†^   - *Explicit* - *Implicit* | 7 | 4 (57)  *1 (14)*  *3 (43)* | 40 | 22 (55)  *6 (15)*  *16 (40)* | 2 | 1 (50)  *0 (0)*  *1 (50)* |
| 7 Switching/ add on | 16 | 12 (75) | 60 | 35 (60) | 5 | 3 (60) |
| 8 Codes | 18 | 5 (28) | 67 | 18 (27) | 6 | 1 (17) |
| 9 Frequency and temporality of codes | 18 | 16 (89) | 67 | 55 (82) | 6 | 6 (100) |
| 10 Care setting | 18 | 13 (72) | 67 | 49 (73) | 6 | 5 (83) |
| 11 Exposure Assessment Window (EAW) | 18 | 18 (100) | 67 | 65 (97) | 6 | 6 (100) |

^†^ When explicitly mentioning an introduction period, a period after the index date is clearly excluded in the exposure risk window. Stating that the follow-up started on the day of the first prescription implies implicitly that there was no induction period. The same reasoning also applies to the extension period.

^‡^ Total number of studies for which this item was applicable.

**Table S5 - Reporting quality of the studies included in this systematic review of the quality of reporting in pharmacoepidemiology stratified per type of database. For each specific item, the number of studies reporting that item is shown.**

|  | Claims (n=41) | | Pharmacy (n=16) | | GP (n=17) | | Hospital (n=18) | |
| --- | --- | --- | --- | --- | --- | --- | --- | --- |
|  | Studies, *n* ^‡^ | Reported, n *(%)* | Studies, *n* ^‡^ | Reported, n *(%)* | Studies, *n* ^‡^ | Reported, n *(%)* | Studies, *n* ^‡^ | Reported, n *(%)* |
| 1 Type of exposure | 41 | 41 (100) | 16 | 16 (100) | 17 | 17 (100) | 18 | 18 (100) |
| 2 Exposure risk window (ERW) | 41 | 39 (95) | 16 | 15 (94) | 17 | 15 (88) | 18 | 9 (50) |
| 3 Induction period ^†^   - *Explicit* - *Implicit* | 41 | 39 (95)  *6 (15)*  *33 (80)* | 15 | 14 (93)  *5 (33)*  *9 (60)* | 17 | 15 (88)  *1 (6)*  *14 (82)* | 18 | 14 (78)  *2 (11)*  *12 (67)* |
| 4 Stockpiling | 20 | 5 (25) | 9 | 0 (0) | 14 | 0 (0) | 6 | 0 (0) |
| 5 Bridging exposure episodes | 17 | 12 (71) | 9 | 3 (33) | 14 | 3 (21) | 6 | 0 (0) |
| 6 Exposure extension ^†^   - *Explicit* - *Implicit* | 18 | 12 (67)  *5 (28)*  *7 (39)* | 9 | 5 (56)  *0 (0)*  *5 (56)* | 15 | 11 (73)  *4 (27)*  *7 (47)* | 9 | 2 (22)  *2 (22)*  *0 (0)* |
| 7 Switching/ add on | 37 | 27 (73) | 14 | 7 (50) | 16 | 10 (63) | 15 | 9 (60) |
| 8 Codes | 41 | 10 (24) | 16 | 11 (69) | 17 | 5 (29) | 18 | 0 (0) |
| 9 Frequency and temporality of codes | 41 | 40 (98) | 16 | 15 (94) | 17 | 15 (88) | 18 | 9 (50) |
| 10 Care setting | 41 | 22 (54) | 16 | 13 (81) | 17 | 17 (100) | 18 | 18 (100) |
| 11 Exposure Assessment Window (EAW) | 41 | 39 (95) | 16 | 16 (100) | 17 | 17 (100) | 18 | 18 (100) |

^†^ When explicitly mentioning an introduction period, a period after the index date is clearly excluded in the exposure risk window. Stating that the follow-up started on the day of the first prescription implies implicitly that there was no induction period. The same reasoning also applies to the extension period.

^‡^ Total number of studies for which this item was applicable.

**Table S6 - Reporting quality of the studies included in this systematic review of the quality of reporting in pharmacoepidemiology stratified per maximum of words allowed. For each specific item, the number of studies reporting that item is shown.**

|  | Short reports | | Original research articles | | | | | |
| --- | --- | --- | --- | --- | --- | --- | --- | --- |
|  | Word limit ≤1500 (n=4) | | Word limit 1500-3000 (n=32) ^§^ | | Word limit 3000-4000 (n=30) ^§^ | | Word limit ≥4000 (n=25) ^§^ | |
|  | Studies, *n* ^‡^ | Reported, n *(%)* | Studies, *n* ^‡^ | Reported, n *(%)* | Studies, *n* ^‡^ | Reported, n *(%)* | Studies, *n* ^‡^ | Reported, n *(%)* |
| 1 Type of exposure | 4 | 4 (100) | 32 | 32 (100) | 30 | 30 (100) | 25 | 25 (100) |
| 2 Exposure risk window (ERW) | 4 | 3 (75) | 32 | 30 (94) | 30 | 25 (83) | 25 | 19 (76) |
| 3 Induction period ^†^   - *Explicit* - *Implicit* | 4 | 3 (75)  *1 (25)*  *2 (50)* | 32 | 30 (94)  *6 (19)*  *24 (75)* | 30 | 26 (87)  *6 (20)*  *20 (67)* | 24 | 22 (92)  *20 (83)*  *2 (8)* |
| 4 Stockpiling | 1 | 0 (0) | 20 | 4 (20) | 13 | 0 (0) | 13 | 1 (7) |
| 5 Bridging exposure episodes | 1 | 0 (0) | 17 | 8 (47) | 13 | 6 (46) | 13 | 4 (31) |
| 6 Exposure extension ^†^   - *Explicit* - *Implicit* | 2 | 1 (50)  *0 (0)*  *1 (50)* | 18 | 10 (56)  *5 (28)*  *5 (28)* | 16 | 9 (56)  *6 (38)*  *3 (19)* | 13 | 7 (54)  *6 (46)*  *1 (7)* |
| 7 Switching/ add on | 4 | 3 (75) | 28 | 16 (57) | 27 | 17 (62) | 22 | 14 (64) |
| 8 Codes | 4 | 1 (25) | 32 | 8 (25) | 30 | 7 (23) | 25 | 8 (32) |
| 9 Frequency and temporality of codes | 4 | 3 (75) | 32 | 28 (88) | 30 | 25 (83) | 25 | 21 (84) |
| 10 Care setting | 4 | 2 (50) | 32 | 24 (75) | 30 | 24 (80) | 25 | 17 (68) |
| 11 Exposure Assessment Window (EAW) | 4 | 4 (100) | 32 | 31 (97) | 30 | 29 (97) | 25 | 24 (96) |

^†^ When explicitly mentioning an introduction period, a period after the index date is clearly excluded in the exposure risk window. Stating that the follow-up started on the day of the first prescription implies implicitly that there was no induction period. The same reasoning also applies to the extension period.

^‡^ Total number of studies for which this item was applicable.

^§^ Word limit per journal, according to the author guidelines of the journals: Annals of Pharmacotherapy 3000; British Journal of Clinical Pharmacology 3000-4000; Drug Safety 6000; European Journal of Clinical Pharmacology 8-10 pages of around 450 words (=4500 words maximum); Pharmacoepidemiology and Drug Safety 3500; and Pharmacotherapy 3500.

**Table S7 - Reporting quality of the studies included in this systematic review of the quality of reporting in pharmacoepidemiology stratified per route of administration (oral/inhaled vs IV/subcutaneous). For each specific item, the number of studies reporting that item is shown.**

|  | Oral/inhaled (n=80) | | IV/subcutaneous (n=11) | |
| --- | --- | --- | --- | --- |
|  | Studies, *n* ^‡^ | Reported, n *(%)* | Studies, *n* ^‡^ | Reported, n *(%)* |
| 1 Type of exposure | 80 | 80 (100) | 11 | 11 (100) |
| 2 Exposure risk window (ERW) | 80 | 71 (89) | 11 | 6 (55) |
| 3 Induction period ^†^   - *Explicit* - *Implicit* | 79 | 73 (92)  *11 (14)*  *62 (78)* | 11 | 8 (72)  *3 (27)*  *5 (45)* |
| 4 Stockpiling | 44 | 5 (11) | 3 | 0 (0) |
| 5 Bridging exposure episodes | 41 | 18 (44) | 3 | 0 (0) |
| 6 Exposure extension ^†^   - *Explicit* - *Implicit* | 43 | 26 (60)  *17 (40)*  *9 (21)* | 6 | 1 (17)  *0 (0)*  *1 (17)* |
| 7 Switching/ add on | 74 | 46 (62) | 7 | 4 (57) |
| 8 Codes | 80 | 23 (29) | 11 | 1 (9) |
| 9 Frequency and temporality of codes | 80 | 71 (89) | 11 | 6 (55) |
| 10 Care setting | 80 | 58 (73) | 11 | 9 (82) |
| 11 Exposure Assessment Window (EAW) | 80 | 78 (98) | 11 | 11 (100) |

^†^ When explicitly mentioning an introduction period, a period after the index date is clearly excluded in the exposure risk window. Stating that the follow-up started on the day of the first prescription implies implicitly that there was no induction period. The same reasoning also applies to the extension period.

^‡^ Total number of studies for which this item was applicable
