# Supplementary material for: Quality of reporting of drug exposure in pharmacoepidemiological studies
Source: Pharmacoepidemiol Drug Saf. 2020 May 11;29(9):1141–50. doi: 10.1002/pds.5020 (PMC7539966; doi:10.1002/pds.5020)
Supplement: Supplementary file 4 — Data S4. Supporting Information. [file PDS-29-1141-s004.docx]

# Supplementary materials - interobserver agreement per item (S4)

| Item | Scores | | | Kappa | Overall agreement * |
| --- | --- | --- | --- | --- | --- |
|  | *Yes* | *No* | *NA* |  |  |
| **1 Type of exposure** | 91 | 0 | 0 | 0,11 | 0,88 |
| **2 Exposure risk window (ERW)** | 77 | 14 | 0 | 0,49 | 0,82 |
| **3 Induction period** | 71 | 9 | 1 | 0,20 | 0,66 |
| **4 Stockpiling** | 5 | 42 | 33 | 0,33 | 0,64 |
| **5 Bridging exposure episodes** | 18 | 26 | 47 | 0,35 | 0,58 |
| **6 Exposure extension** | 27 | 22 | 42 | 0,28 | 0,52 |
| **7 Switching/ add on** | 50 | 31 | 10 | 0,33 | 0,61 |
| **8 Codes** | 24 | 67 | 0 | 0,75 | 0,90 |
| **9 Frequency and temporality of codes** | 77 | 14 | 0 | 0,35 | 0,76 |
| **10 Care setting** | 67 | 24 | 0 | 0,43 | 0,78 |
| **11 Exposure Assessment Window (EAW)** | 89 | 2 | 0 | 0,23 | 0,93 |

* calculated as the proportion of publications agreed upon directly.
